# A dimensional measure of safety behavior: A non-dichotomous assessment of costly avoidance in human fear conditioning

**DOI:** 10.1007/s00426-021-01490-w

**Published:** 2021-03-04

**Authors:** Alex H. K. Wong, Andre Pittig

**Affiliations:** 1 Department of Psychology (Biological Psychology, Clinical Psychology, and Psychotherapy), University of Würzburg, Würzburg, Germany; 2 Center of Mental Health, University of Würzburg, Würzburg, Germany

## Abstract

Safety behavior prevents the occurrence of threat, thus it is typically considered adaptive. However, safety behavior in anxiety-related disorders is often costly, and persists even the situation does not entail realistic threat. Individuals can engage in safety behavior to varying extents, however, these behaviors are typically measured dichotomously (i.e., to execute or not). To better understand the nuances of safety behavior, this study developed a dimensional measure of safety behavior that had a negative linear relationship with the admission of an aversive outcome. In two experiments, a Reward group receiving fixed or individually calibrated incentives competing with safety behavior showed reduced safety behavior than a Control group receiving no incentives. This allowed extinction learning to a previously learnt warning signal in the Reward group (i.e., updating the belief that this stimulus no longer signals threat). Despite the Reward group exhibited extinction learning, both groups showed a similar increase in fear to the warning signal once safety behavior was no longer available. This null group difference was due to some participants in the Reward group not incentivized enough to disengage from safety behavior. Dimensional assessment revealed a dissociation between low fear but substantial safety behavior to a safety signal in the Control group. This suggests that low-cost safety behavior does not accurately reflect the fear-driven processes, but also other non-fear-driven processes, such as cost (i.e., engage in safety behavior merely because it bears little to no cost). Pinpointing both processes is important for furthering the understanding of safety behavior.

**Supplementary Information:**

The online version contains supplementary material available at 10.1007/s00426-021-01490-w.

## Introduction

When confronting a threatening object or situation, organisms often engage in safety behavior that aims to prevent the occurrence of a threatening outcome. In this regard, safety behavior is adaptive given that it effectively prevents harm. However, safety behavior in anxiety-related disorders is oftentimes maladaptive as it is out of proportion to realistic threat, persistent in the absence of threat, and linked to impairments in everyday functioning (Mendlowicz & Stein, 2000; Olatunji, Cisler, & Tolin, 2007). For instance, an individual with social anxiety disorder may actively avoid pauses when conversing with others to avoid looking anxious, thereby reducing the perceived threat of being negatively evaluated (Kim, 2005).

Fear and avoidance conditioning provide a well-established laboratory model for examining fear-related safety behavior, which usually combines both Pavlovian fear acquisition and instrumental learning. During Pavlovian fear acquisition, an initially neutral conditioned stimulus (CS+) is repeatedly paired with an aversive unconditioned stimulus (US). In a subsequent instrumental learning phase, participants learn that performing a designated response during CS+ presentation prevents the US. This response is referred to as “US-avoidance” given that it prevents the US but does not terminate CS+ presentation (see Pittig, Wong, Glück, & Boschet, 2020). Performing US-avoidance was found to reduce conditioned fear responses to the CS+ (e.g., Lovibond, Saunders, Weidemann, & Mitchell, 2008; Morriss, Chapman, Tomlinson, & van Reekum, 2018; Pittig, 2019), analogous to the reduction in anxiety after engaging in safety behavior when confronting fear-related stimuli or situations. This reduction in anxiety aligns with a propositional Expectancy model (Lovibond, 2006). One prediction this model makes is a decrease in conditioned fear responses to the CS+ once US-avoidance is performed, since the US is not anticipated anymore (cf. Two-factor theory; Mowrer, 1939).

Excessive engagement with safety behavior has been found to preserve maladaptive threat beliefs. Empirical studies found that engagement in US-avoidance during extinction led to heightened fear responses to the CS+ when avoidance availability was removed (Lovibond, Mitchell, Minard, Brady, & Menzies, 2009; Pittig, 2019; Rattel, Miedl, Blechert, & Wilhelm, 2017; Volders, Meulders, Peuter, Vervliet, & Vlaeyen, 2012; Volders, Meulders, Peuter, & Vlaeyen, 2015). This pattern was referred to as ‘protection from extinction’: threat belief to the CS+ was intact, because the absence of an US during extinction was attributed to US-avoidance, therefore, preventing extinction learning to take place. The findings of these studies aligned with clinical observations in which individuals with clinical anxiety excessively engage in safety behaviors showed resistance to exposure therapy or relapse after apparent successful treatment (Helbig-Lang & Petermann, 2010; Salkovskis, Clark, & Gelder, 1996; Wells et al., 1995).

Although the aforementioned studies provided valuable insights into the mechanisms of avoidance and safety behavior, traditional paradigms have recently been criticized for examining low-cost safety behavior (Krypotos, Vervliet & Engelhard, 2018). Low-cost safety behavior refers to US-avoidance that requires minimal cost or effort (e.g., merely pressing a button). Low-cost US-avoidance arguably does not resemble pathological safety behavior in anxiety-related disorders: safety behavior in anxiety-related disorders often bears a cost. In light of this, more recent laboratory studies have incorporated a competing reward to safety behavior (e.g., Claes, Karos, Meulders, Crombez, & Vlaeyen, 2014; Pittig, 2019; Rattel et al., 2017; van Damme, van Ryckeghem, Wyffels, van Hulle, & Crombez, 2012). That is, an appetitive outcome that motivates behavioral approach to the fear-related stimulus (i.e., approach-avoidance conflict), rendering avoidance costly (i.e., missing out the competing reward). A common finding from these studies is that healthy individuals showed less US-avoidance with the presence of a competing reward. Furthermore, preliminary studies showed that a decrease in costly US-avoidance did not reduce conditioned fear to the CS+; instead, the competing reward per se acts as an incentive for behavioral approach (fear-opposite actions; Pittig & Dehler, 2019). This incentive for non-avoidance response has been found to facilitate extinction learning (Pittig, 2019; Rattel et al., 2017) and subsequently alleviated the effect of protection from extinction (Pittig, 2019). Collectively, these findings suggest that incorporating a cost in US-avoidance may provide a useful testbed for investigating pathological safety behavior.

Another criticism refers to safety behavior commonly investigated in a dichotomous manner. That is, it is either performed or not performed. Little research has focused on examining the magnitude or the extent of engagement in safety behavior. It has been suggested that patients sometimes only engage in safety behavior to a certain degree (see Krypotos et al., 2018; Telch & Lancaster, 2012). For example, an individual with social anxiety may rather converse to a lesser extent in a group discussion. While this safety behavior is believed to prevent the perceived threatening outcome (e.g., appear unintelligent in the conversation) to a certain extent, the individual could still contribute to the group discussion to some extent (obtaining the competing reward; Kashdan, Elhai, & Breen, 2008). In this regard, safety behavior is oftentimes not a dichotomous behavior, but can be seen as a balance of keeping threat at a subjectively acceptable level while limiting its costs (cf. Schlund et al., 2016). A dimensional measure of safety behavior is thus arguably more sensitive to measure the different degrees of safety behavior. Furthermore, a dichotomous measure usually results in a ceiling effect with little variability in responding (i.e., most participants fully engaging in safety behavior). The lack of variability decreases the sensitivity to examine individual differences or risk factors modulating the acquisition of safety behavior, such as trait anxiety, intolerance of uncertainty, and anxiety sensitivity (see Lonsdorf & Merz, 2017; Pittig et al., 2020). A dimensional measure may also provide important theoretical insights. For example, it may be a more sensitive test for predictions of the Expectancy model (Lovibond, 2006). Specifically, the model assumes that a higher degree of safety behavior predicts lower conditioned fear to the CS+, or that the degree of US-avoidance during extinction predicts the magnitude of protection from extinction. In sum, a dimensional measure of US-avoidance potentially provides some advantages over the traditional dichotomous measure.

Non-fear conditioning studies have already entertained the idea of measuring defensive behaviors (behaviors that entail escape or avoidance, see Krypotos et al., 2018) dimensionally. For instance, the “Pac-man” task (Mobbs et al., 2007, 2009) and the foraging task (Bach et al., 2014) operationalized defensive behaviors as the distance between an individual’s avatar and a virtual predator. Behavioural Approach Task serves as another paradigm to measure avoidance dimensionally (e.g., Shiban, Pauli & Mühlberger, 2013). In contrast, little research has measured avoidance dimensionally, especially US-avoidance, within a fear conditioning framework. Flores, Lopez, Vervliet and Cobos (2018) pioneered a novel continuous measure of US-avoidance in a fear conditioning paradigm. Participants were presented with a CS+ of 20 s, in which an US was randomly delivered between 8 and 11 s after CS+ onset. Participants were informed that pressing a designated key during CS+ presentation may prevent the US, but only keypresses within 1 s before US onset could effectively prevent the US. Given the uncertain timing of US administration, participants were encouraged to press the designated key as many times as they wanted during CS+ presentation. Therefore, this study elegantly examined the magnitude of safety behavior as a function of the level of US uncertainty. However, Flores et al. (2018) did not incorporate a competing reward for US-avoidance, therefore, may not fully tap into the pathological domain of safety behavior. Furthermore, this novel procedure had no room for the measure of conditioned fear to the CS+ after safety behavior had been performed.

In two experiments, we, therefore, developed a dimensional measure of US-avoidance that catered the inclusion of a competing reward and the measurement of conditioned fear after US-avoidance responses, within a fear conditioning framework. We measured US-avoidance using a visual analog scale (0% = complete non-avoidance, 100% = complete avoidance), in which the US-avoidance was negatively proportional to US administration and the competing reward. Therefore, we could examine the role of competing reward in the degree of US-avoidance engagement. Immediately after US-avoidance was made, self-reported US expectancy ratings and skin conductance responses (SCRs) to the CS were measured to reflect the cognitive and physiological aspects of conditioned fear, respectively. This study had two overarching goals: using a dimensional US-avoidance measure to (1) replicate findings on the dynamics between US-avoidance, conditioned fear, and competing reward and (2) testing predictions of the Expectancy model. For the first goal, we aimed to replicate findings on (1a) the dynamics between US-avoidance, conditioned fear, and competing reward during the acquisition of US-avoidance. Specifically, engaging in low-cost US-avoidance decreases conditioned fear, however, an introduction of competing reward reduces US-avoidance, resulting in an increase in conditioned fear; (1b) the dynamics between US-avoidance, competing reward, and extinction learning. Specifically, competing reward reduces US-avoidance, thereby enables extinction learning when US administration is discontinued; and (1c) competing reward reduces protection from extinction. For the second goal, we assumed that a dimensional measure of US-avoidance is more sensitive to predict subsequent levels of conditioned fear. More specifically, the Expectancy model predicts (2a) a negative linear relationship between US-avoidance and conditioned fear. Specifically, an increase in US-avoidance predicts a greater decrease in conditioned fear to the CS+ than the CS–; and (2b) a positive linear relationship between US-avoidance and protection from extinction. This means, an increase in US-avoidance during extinction predicts a stronger protection from extinction effect.

## Method

### Participants

Psychology undergraduates or residents from Würzburg were recruited and received either partial course credit or 9€ for participation. A total of 100 participants were recruited. Half of them were randomly assigned to the Reward group, in which a competing reward was introduce alongside US-avoidance in certain phases. The remaining half were assigned to the Control group with no introduction of competing reward. We carried out data analysis within a linear mixed model framework (see Scoring and analysis), and did not carry out a power analysis for two reasons. First, prior data for power analysis within a linear mixed model is still tentative for an appropriate evaluation of power calculations, especially for the detection of interaction effects (see Mathieu, Aguinis, Culpepper, & Chen, 2012). Second, there is no consensus in the methods for power detection in a linear mixed model, whereas the different methods (e.g., different estimation methods, different types of variance–covariance structures for errors) for power analyses could yield different power calculations (Bahçecitapar, 2018). Alternatively, we followed the sample size of a previous study that also examined the impact of competing reward on extinction learning and protection from extinction (Pittig, 2019). The study was approved by the Ethics Committee of the Institute of Psychology at the University of Würzburg (GZ 2018–25) in accordance to the Declaration of Helsinki.

### Apparatus and materials

**Fig. 1:**
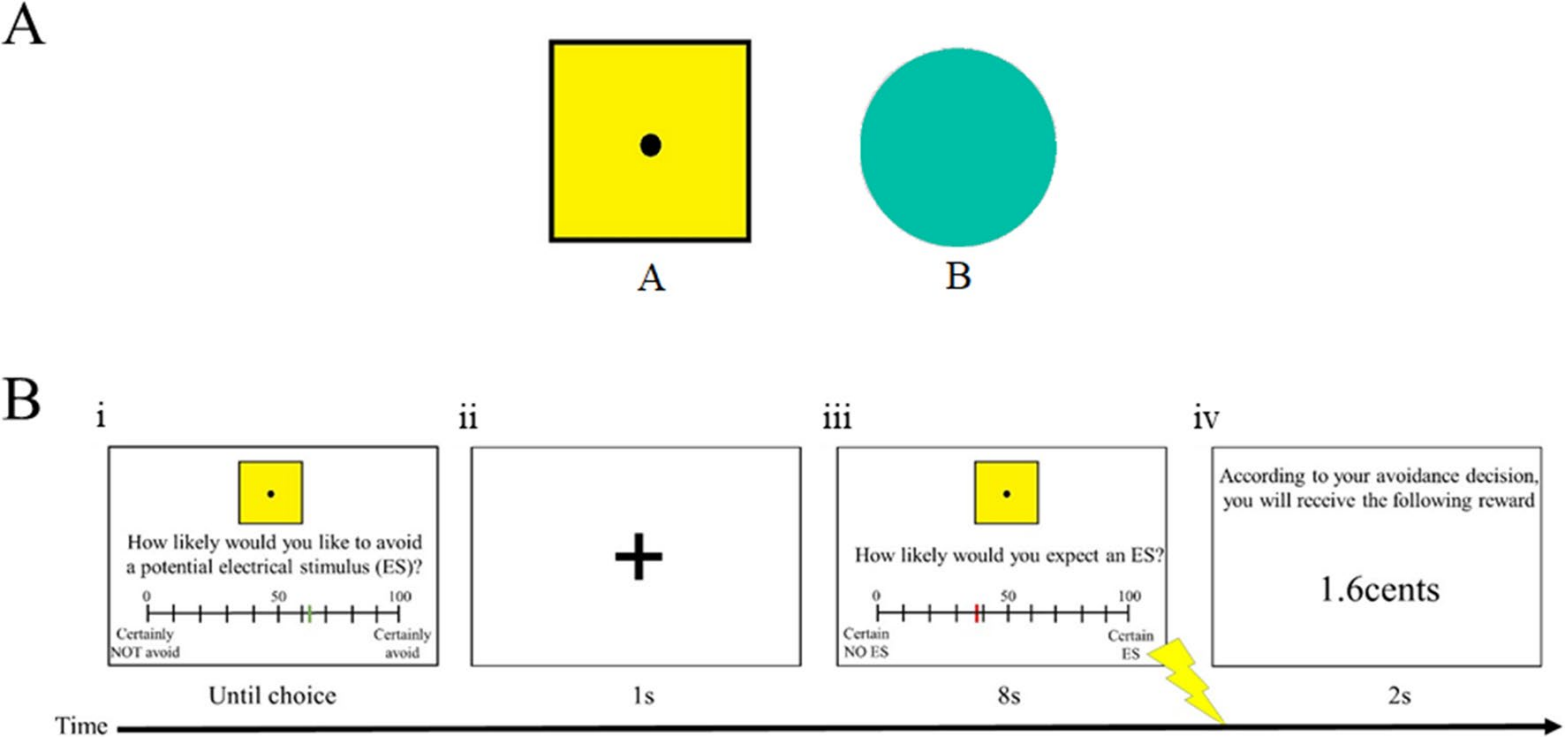
**a** Stimuli used in the two experiments. Stimulus A served as CS+ while stimulus B served as CS−. **b** Example of the trial structure during the US-avoidance-reward phase. (i) Participants had to make their US-avoidance. (ii) A fixation cross appeared on the screen for 1 s. (iii) The CS was presented again with the US expectancy scale for 8 s. An electrical US would be delivered immediately after CS offset depending on the CS type and the US-avoidance made. (iv) The reward feedback appeared on the screen for 2 s for the Reward group, while the Control group received a blank screen for 2 s

Two geometric shapes were used as the CS+ and CS− (see Fig. 1a). One stimulus was a yellow square (9.5 × 9.5 cm) with black outline containing a black dot in the center of the square. The other stimulus was an aqua color circle with a radius of 5.75 cm. The hue of the circle stimulus was 0.479 (hue saturation value model, a saturation of 1 and brightness of 0.75). The two stimuli were of different visual dimensions to minimize perceptual generalization. The square stimulus always served as the CS+ and the circle stimulus always served as the CS–. The stimuli were generated by a computer equipped with Presentation software (Neurobehavioral Systems), which also recorded the US expectancy ratings and the US-avoidance.

### Procedure

After providing written informed consent, participants filled in the German version of DASS-21 (Lovibond & Lovibond, 1995; Nilges & Essau, 2015). The DASS-21 is a short version of the original DASS (Depression Anxiety Stress scale), which measures three psychometric constructs, namely depression, anxiety and stress. DASS-21 has been shown to validly measure and discriminate between these three constructs (Antony, Bieling, Cox, Enns, & Swinson, 1998; Henry & Crawford, 2005). Next, the electrical stimulation electrodes and skin conductance electrodes were attached. Skin conductance electrodes filled with isotonic gel were attached to the hypothenar muscles on the palm of the non-dominant hand. Skin conductance data were measured by BrainVision recorder via two Ag/AgCl electrodes at a sampling rate of 1000 Hz. Participants were then led through a workup procedure in which they selected a level of US intensity that was ‘definitely uncomfortable but not painful’. The electrical US consisted of 125 pulses separated by 5 ms generated by a DS7A Digitimer stimulator. The US was delivered through a bar electrode attached to the wrist of participants’ non-dominant hand.

As shown in Table 1, the experiment consisted of five phases: Pavlovian fear acquisition training, US-avoidance acquisition, US-avoidance-reward, Pavlovian extinction and Test. The Reward group and the Control group only differed in the US-avoidance-reward phase and the Pavlovian extinction phase, in which a competing reward was introduced in the Reward group.

**Table 1:** Design of Experiment 1 and 2

	Pavlovian fear acquisition training	US-avoidance acquisition	US-avoidance-reward	Pavlovian extinction	Test
Reward group	A+ (8)	A*(+) (4)	A*(+) [€] (8)	A*− [€] (12)	A− (4)
	B− (8)	B*− (4)	B*− [€] (8)	B*− [€] (12)	B− (4)
Control group	A+ (8)	A*(+) (4)	A*(+) [NR] (8)	A*− [NR] (12)	A− (4)
	B− (8)	B*− (4)	B*− [NR] (8)	B*− [NR] (12)	B− (4)

Letters A and B refer to conditioned stimuli; + indicates electrical US presentation; − indicates electrical US omission; * indicates the availability of US-avoidance; + in parentheses indicates that the presentation of an electrical US depends on US-avoidance; € indicates competing reward, NR indicates no competing reward; Number in parentheses indicate the number of trials

In all five phases, the CSs were presented at the center of the screen for 8 s. During each CS presentation, participants were instructed to indicate their US expectancy using a visual analog scale that appeared at the bottom of the screen. The scale ranged from 0 to 100% with increments of 1% (0% indicates certain no electrical US and 100% indicates certain electrical US), and the cursor always started at 50%. The expectancy scale co-terminated with the CS. The SCRs were measured online throughout the experiment. US-avoidance responses were only available in the US-avoidance acquisition, US-avoidance-reward and Pavlovian extinction phases. During these three phases, a CS appeared alongside an US-avoidance visual analog scale, and participants were prompted to indicate their degree of US-avoidance. The scale ranged from 0 to 100% with increments of 1%, and the cursor always started at 50%. Once participants indicated the degree of US-avoidance, the CS and the US-avoidance scale co-terminated. Following a 1 second fixation cross, the same CS was presented for 8 s alongside the expectancy scale (see Fig. 1b). The intertrial interval (ITI) was randomized between 15 and 18 s in all five phases.

#### Pavlovian fear acquisition training

Eight trials of stimulus A and eight trials of stimulus B were presented as the CSs. Participants were prompted to indicate their US expectancy ratings using the expectancy scale during the 8 s of CS presentation. Stimulus A (CS+) was fully reinforced (i.e., 100% reinforcement rate) while stimulus B (CS−) was never reinforced.

#### US-avoidance acquisition

Participants were informed that they could prevent a potential US that followed the geometric shapes. The CSs were presented along with the US-avoidance scale. Participants were instructed that if an US would follow the geometric shape, the avoidance they made would be negatively proportional to the chance of receiving an US. For instance, if an individual participant made an US-avoidance of 70%, then there would be a 30% chance of receiving an US if it would had followed the CS. After participants made their US-avoidance, they were prompted to indicate their US expectancy ratings to the same CS. This phase consisted of four CS+ and four CS– trials. The CS+ was reinforced according to participants’ US-avoidance, while the CS− was never reinforced regardless of participants’ US-avoidance.

#### US-avoidance-reward

Before this phase, the Reward group was instructed that each trial would be accompanied by a monetary reward of a maximum of 4 cents. The amount of reward was inversely proportional to the US-avoidance. For instance, an US-avoidance response of 60% would result in a gain of 40% of the maximum reward (i.e., 1.6 cents). Participants in the Reward group were instructed that all reward gained would be paid after the experiment. The Control group, however, did not receive such instructions. The trial structure of this phase was the same as the previous phase, with the exception that the Reward group receiving a reward feedback for 2 s after US expectancies were made, whereas the Control group received a blank screen for 2 s after US expectancies were made (see Fig. 1b).

#### Pavlovian extinction

This phase continued seamlessly from the previous phase without any interruption of instructions in both groups. Participants were not interrupted by any instructions so that participants who engaged in US-avoidance excessively (e.g., the Control group) might continue to attribute the absence of an US to their safety behavior, while those motivated to disengage from US-avoidance (e.g., the Reward group) might have the opportunity to learn the CS–NoUS contingency. This allowed potential group differences in protection from extinction to be observed (see Lovibond et al., 2008; Pittig, 2019; Rattel et al., 2017). The trial structure was similar to the previous phase. However, no USs were presented regardless of CS type.

#### Test

Both groups were instructed that the option of avoiding a potential electrical US was removed. The CSs were presented for four trials each and participants were prompted to rate their US expectancies. None of the CSs were reinforced.

#### Scoring and analysis

Although skin conductance was measured online throughout the experiment, only skin conductance data recorded during the 8 s CS presentation (i.e., when participants were indicating their US expectancy ratings) was included for analysis. We applied a 1 Hz high-pass filter to remove high-frequency noise and a notch filter (50 Hz) to the skin conductance data. Next, we calculated the SCRs by finding the difference between the maximum response and the corresponding trough in the interval of 1 s after CS onset to CS offset (see Pineles, Orr, & Orr, 2009). The resulting SCRs were then square root transformed to reduce skewness (Boucsein et al., 2012).

We analysed all data within a linear mixed model framework. As a manipulation check, we first analysed whether differential conditioned fear (higher expectancy ratings and stronger SCRs to the CS+ than the CS−) was acquired in Pavlovian fear acquisition training. For this purpose, expectancy ratings or SCRs served as the dependent variable (i.e., expectancy ratings and SCRs served as the dependent variable in separate models). Fixed effects included CS type (CS− served as a reference trial type), Group (Control group served as a reference group) and Trial (first trial served as a reference trial, represented by a value of 0). Participants served as a random effect. All effects and interactions of the fixed effects were evaluated, and were applied to all the models described below. Importantly, Hayes, Glynn and Huge (2012) argued that when all effects coexist in the same model, the lower order effects represent simple effects of the higher order interaction. Therefore, we analysed the main effects, the two-way interactions, and the three-way interactions in separate models.

Different models were constructed to test the hypotheses derived from the two overarching goals:

- The dynamics between US-avoidance, conditioned fear, and competing reward during the acquisition of US-avoidance

We analysed US-avoidance, expectancy ratings and SCRs as dependent variables between the US-avoidance acquisition and US-avoidance-reward phases. CS type (CS− served as a reference trial type), Phase (US-avoidance acquisition phase served as a reference phase) and Group (Control group served as a reference group) served as fixed effects. Participants served as a random effect. These models captured the changes in US-avoidance and conditioned fear from US-avoidance acquisition to US-avoidance-reward and tested for potential group differences.

1. The dynamics between US-avoidance, competing reward, and extinction learning

During Pavlovian extinction, US-avoidance, expectancy ratings, or SCRs served as the dependent variable. CS type (CS− served as a reference trial type), Group (Control group served as a reference group) and Trial (the first extinction trial served as a reference trial) served as fix effects, whereas participants were treated as a random effect. These models captured the change in US-avoidance and conditioned fear across extinction and examined if there were any group differences.

1. Competing reward reduces protection from extinction

To test for the magnitude of protection from extinction effect, we compared the expectancy ratings and SCRs on the last extinction trial to the first test trial. In these models, expectancy ratings or SCRs served as the dependent variable. CS type (CS− served as a reference trial type), Trial (extinction trial served as a reference trial) and Group (Control group served as a reference group) served as fixed effects, whereas participants served as a random effect.

- A negative linear relationship between US-avoidance and conditioned fear

We tested whether an increase in US-avoidance would predict a decrease in conditioned fear, especially to the CS+ , averaged across the US-avoidance acquisition and US-avoidance-reward phases. Expectancy ratings or SCRs were the dependent variable. US-avoidance (a continuous predictor) and CS type (CS− served as a reference trial type) served as fixed effects, whereas participants served as a random effect. Phase was not served as a fixed effect given that we had no a priori hypotheses on how competing reward would *directly* determine conditioned fear per se (see Pittig & Dehler, 2019).

2b. A positive linear relationship between US-avoidance and protection from extinction

We tested whether the degree of US-avoidance on the last extinction trial predicted the magnitude of conditioned fear on the first test trial. Expectancy ratings or SCRs were the dependent variable. Fixed effects included US-avoidance (a continuous predictor) and CS type (CS– served as a reference trial type), whereas participants served as a random effect.

In all the aforementioned linear mixed models, the degree of significance was reported with Satterthwaite approximation for degrees of freedom (Satterthwaite, 1941). All analyses were carried out using R (R Core Team, 2020) with lmer package (Bates, Mächler, Bolker & Walker, 2015).

### Results

Statistical analyses were restricted to participants who had acquired conditioned fear to the CS+ , that is, participants who demonstrated differential conditioned responding to the CSs in their US expectancy ratings. Differential conditioning between CS+ and CS− was defined by an average difference of at least 50 in the last four trials of Pavlovian fear acquisition training. Two participants (1 in each group) were excluded based on this criterion. We did not include an inclusion criterion for US-avoidance acquisition given that some participants may also showed high degree of US-avoidance to the CS– due to the low-cost to execute US-avoidance during US-avoidance acquisition (cf. Lommen, Engelhard, & van den Hout, 2010). In sum, a total of 98 participants (49 in each group) were included for analyses.^1^ Table S1 (see Supplementary Materials) shows the demographic and DASS-21 data. No group differences were observed. Only critical effects involving CS type or Group were reported, while any other effects were reported in the Supplementary Materials.

#### Pavlovian fear acquisition training

**Fig. 2:**
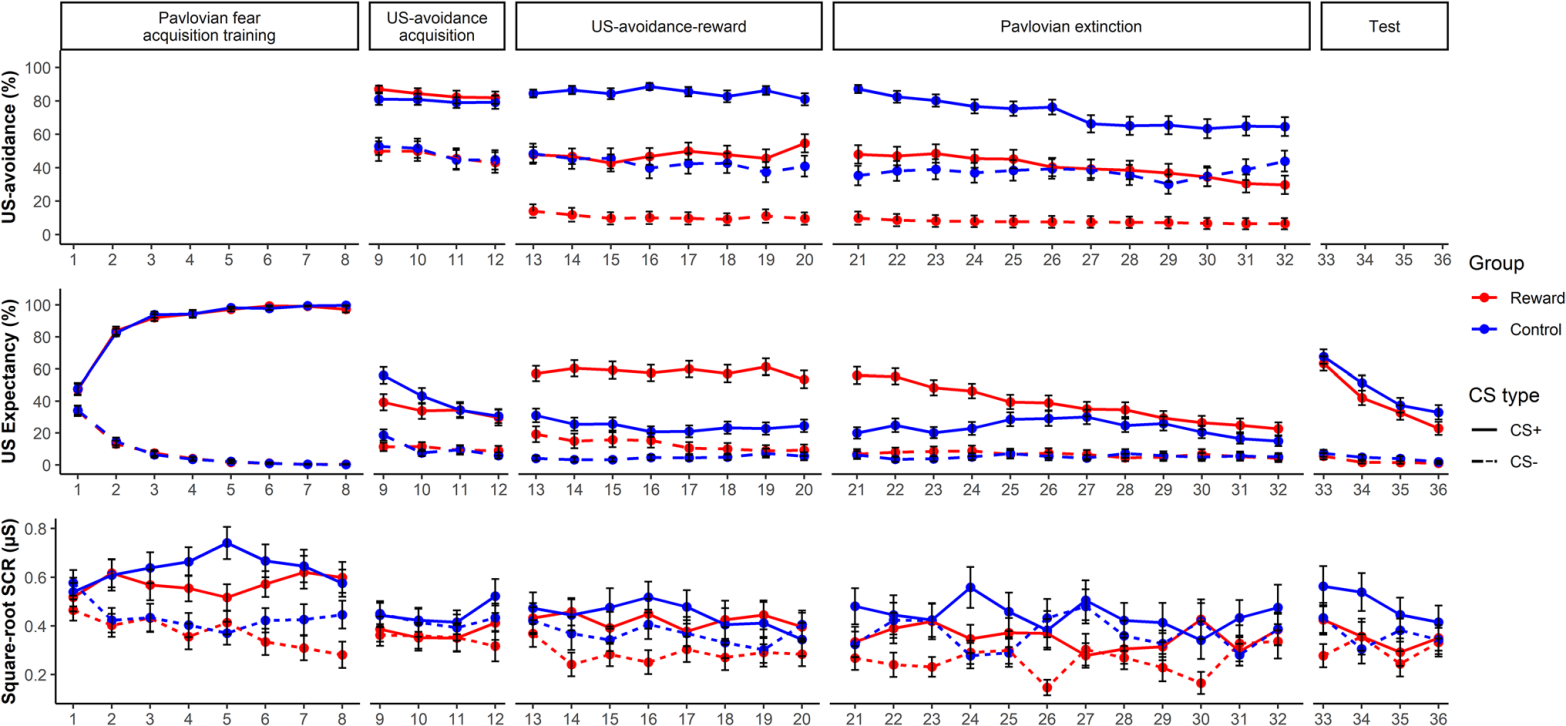
US-avoidance (top panel), US expectancy ratings (middle panel), and square-root SCRs (bottom panel) across all phases between Reward and Control groups in Experiment 1. See the color version of this figure online

Figure 2 shows the US-avoidance, US expectancy ratings and SCRs to the CSs across the different phases. The results of this phase are briefly summarized below (see Supplementary Materials for the detailed analyses). Collectively, both groups acquired differential Pavlovian conditioned fear to the CSs without any group differences.

#### Hypothesis 1a: the dynamics between US-avoidance, conditioned fear and competing reward during the acquisition of US-avoidance

##### US-avoidance

Only the interactions are reported here (see Supplementary Materials for the detailed analyses). We observed a significant Group × Phase interaction, *b*_Group × Phase_ = − 35.95, SE = 2.20, *p* < 0.001, suggesting that when CS type was at its reference level (the CS–), the Reward group showed a larger decrease in US-avoidance in US-avoidance-reward than US-avoidance acquisition. In other words, the Reward group exhibited a decrease in US-avoidance to the CS– when competing reward was introduced, whereas the Control group, which did not receive any competing reward, maintained a relatively high US-avoidance to the CS–. We also observed a CS type × Phase interaction, *b*_CS type × Phase_ = 5.40, SE = 2.20, *p* = 0.014, suggesting when the Group effect was at its reference level (the Control group), the difference in US-avoidance between the CS+ and the CS– was greater in US-avoidance-reward than US-avoidance acquisition. In other words, the Control group showed larger differential US-avoidance to the CSs in the transition from the US-avoidance acquisition phase to the US-avoidance-reward phase. Critically, compared to the Control group, the Reward group exhibited a larger decrease in differential US-avoidance to the CSs, *b*_CS type × Group × Phase_ = − 10.23, SE = 4.39, *p* = 0.020. This means, the introduction of a competing reward systematically reduced US-avoidance more to the CS+ than the CS−.

##### Expectancy ratings and SCRs

Unexpectedly, the Control group showed larger differential expectancy ratings to the CSs than the Reward group in US-avoidance acquisition, *b*_CS × Group_ = 14.96, SE = 1.99, *p* < 0.001, despite both groups showed similar differential US-avoidance to the CSs in the same phase. Compared to the Control group, the Reward group showed a larger increase in expectancy ratings to the CS− when transiting across the two phases, *b*_Group × Phase_ = 24.60, SE = 2.11, *p* < 0.001. This was presumably due to the decrease in US-avoidance to the CS– in the Reward group during the transition across the two phases. Importantly, compared to the Control group, the Reward group showed a larger increase in expectancy ratings to the CS+ than the CS– when transiting across the two phases, *b*_CS type × Group × Phase_ = 32.20, SE = 4.17, *p* < 0.001. This increase in expectancy ratings to the CS+ in the Reward group was presumably due to the decrease in US-avoidance when a competing reward was introduced.

Unlike the expectancy ratings, the SCRs revealed a relatively irregular pattern. The Control group showed stronger differential responding to the CSs in US-avoidance-reward than in US-avoidance acquisition, *b*_CStype × Phase_ = 0.078, SE = 0.030, *p* = 0.001. No other effects nor interactions reached significance (smallest *p* = 0.205).

Collectively, the introduction of a competing reward reduced US-avoidance more to the CS+ than the CS−. Expectancy ratings to the CS+ increased more strongly in the Reward group than the Control group during the transition from US-avoidance acquisition to US-avoidance-reward, because the Reward group was incentivized to engage in US-avoidance to a lesser degree. Interestingly, the Control group showed a higher degree of US-avoidance to the CS– compared to the Reward group.

#### Hypothesis 1b: The dynamics between US-avoidance, competing reward, and extinction learning

##### US-avoidance

A significant main effect of CS type, *b*_CStype_ = 33.80, SE = 0.94, *p* < 0.001, suggested that on the first extinction trial, the Control group showed a greater degree of US-avoidance to the CS+ than the CS−. This differential US-avoidance to the CSs in the Control group decreased across extinction trials, confirmed by a significant interaction between CS type and Trial, *b*_CStype × Trial_ = − 1.90, SE = 0.27, *p* < 0.001. However, this interaction did not further interact with Group, *b*_CStype × Trial × Group_ = 0.73, SE = 0.54 *p* = 0.177, suggesting that there was no evidence for any group differences in the decrease in differential US-avoidance to the CSs. Interestingly, we observed a significant main effect of Group, *b*_Group_ = − 30.92, SE = 5.46, *p* < 0.001, suggesting that on the first extinction trial, the Reward group showed lower US-avoidance to the CS− compared to the Control group. This effect failed to interact with Trial, *b*_Group × Trial_ = 0.014, SE = 0.27, *p* = 0.957, suggesting no evidence of this pattern to change across extinction trials. In other words, this heightened US-avoidance to the CS− in the Control group remained relatively stable across extinction. No other effects reached significance (smallest *p* = 0.177).

##### Expectancy ratings and SCRs

On the first extinction trial, the Control group showed higher expectancy ratings to the CS+ than the CS–, confirmed by a main effect of CS type, *b*_CS type_ = 24.73, SE = 0.84, *p* < 0.001. This effect interacted with Group, *b*_CS type × Group_ = 13.68, SE = 1.63, *p* < 0.001, suggesting that the differential ratings to the CSs on the first extinction trial was larger in the Reward group compared to the Control group. Importantly, this group difference in differential ratings to the CSs decreased across extinction trials, *b*_CS type × Group × Trial_ = − 2.34, SE = 0.47, *p* < 0.001, presumably driven by the decrease in expectancy ratings to the CS+ in the Reward group. However, despite the Reward group showed a greater decrease in expectancy ratings to the CS+ compared to the Control group, the latter still exhibited a larger decrease in ratings to the CS+ than the CS− across extinction trials, *b*_CS type×Trial_ = − 1.65, SE = 0.24, *p* < 0.001. The group differences in expectancy ratings to the CS– also decreased across extinction trials, *b*_Group×Trial_ = − 1.54, SE = 0.24, *p* < 0.001. In sum, these patterns suggested that extinction learning to the CS+ took place in the Reward group, whereas the Control group showed very limited extinction learning.

For the SCRs, the Control group showed stronger responding to the CS+ than the CS− on the first extinction trial, *b*_CS type_ = 0.085, SE = 0.016, *p* < 0.001. No other effects reached significance (smallest *p* = 0.054).

Collectively, the Reward group showed less US-avoidance to the CS+ than the Control group, which enabled extinction learning to the CS+. Extinction learning in the Reward group, however, was only observed in the expectancy ratings, but not in the SCRs. Similar to the previous phase, the Control group continued to exhibit a higher degree of US-avoidance to the CS– compared to the Reward group.

#### Hypothesis 1c: competing reward reduces protection from extinction

##### Expectancy ratings and SCRs

The Control group showed higher expectancy ratings to the CS+ than the CS– on the last extinction trial, supported by a main effect of CS type, *b*_CS type_ = 36.63, SE = 2.56, *p* < 0.001. These differential ratings to the CSs increased from the last extinction trial to the first test trial, *b*_CS type × Trial_ = 45.05, SE = 4.37, *p* < 0.001. This indicated a protection from extinction effect in the Control group. Although the Reward group showed a smaller increase in differential ratings to the CSs from Pavlovian extinction to test (i.e., weaker protection from extinction effect) when compared to the Control group, this three-way interaction did not reach significance, *b*_CS type × Trial × Group_ = − 10.84, SE = 8.72, *p* = 0.215. For the SCR data, the Control group showed stronger responding to the CS+ than the CS- on the last extinction trial, *b*_CS type_ = 0.10, SE = 0.044, *p* = 0.020. No effects in the SCR data reached significance (smallest *p* = 0.081).

#### Hypothesis 2a: A negative linear relationship between US-avoidance and conditioned fear

**Fig. 3:**
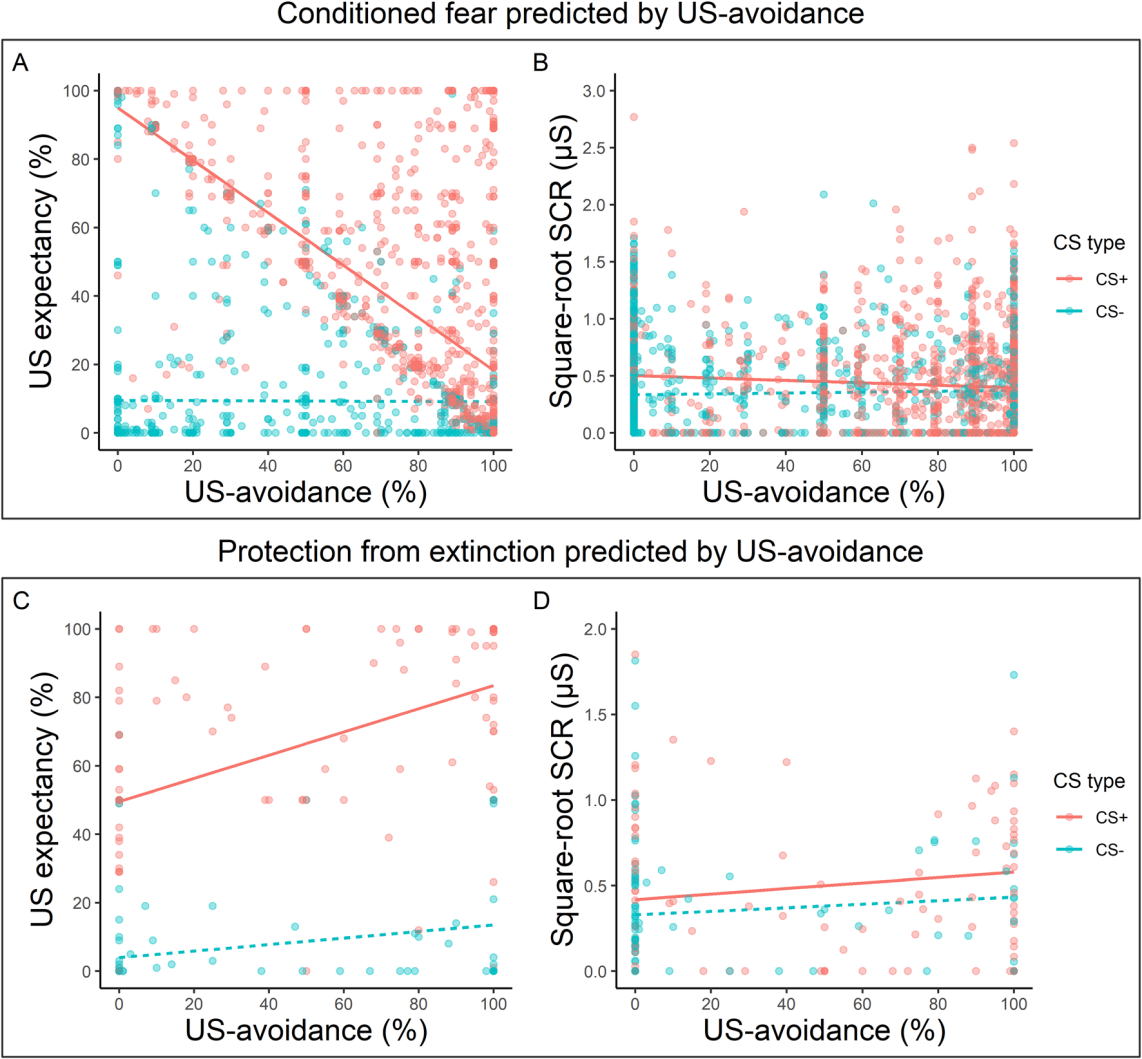
Relationship between US-avoidance and conditioned fear during different phases in Experiment 1. Top Panel. US-avoidance predicts **a** US expectancy ratings and **b** square-root SCRs to the CS+ and the CS– during US-avoidance acquisition and US-avoidance-reward. Bottom Panel. US-avoidance on the last extinction trial predicting **c** US expectancy and **d** square-root SCRs on the first test trial. Red dots represent responding to the CS+ whereas green dots represent responding to the CS−. Darker color indicates more overlapping data points. The lines represent the line of best fit for each CS for visual aid. See the color version of this figure online

Overall, an increase in US-avoidance decreased expectancy ratings to the CS+ more than the CS– (Fig. 3a), confirmed by a significant interaction between CS type and Avoidance, *b*_CS type×Avoidance_ = − 0.70, SE = 0.026, *p* < 0.001.

For the SCRs (Fig. 3b), an increase in US-avoidance led to a greater decrease in responding to the CS+ compared to the CS−, *b*_CS type×Avoidance_ = − 0.0015, SE = 0.00043, *p* < 0.001.

Collectively, an increase in US-avoidance predicted a greater decrease in conditioned fear to the CS+ than the CS−, as indicated in both expectancy ratings and SCRs.

#### Hypothesis 2b: A positive linear relationship between US-avoidance and protection from extinction

Collapsed across the two groups, an increase in US-avoidance on the last extinction trial significantly predicted a larger increase in expectancy ratings for the CS+ than the CS− on the first test trial (Fig. 3c), confirmed by a significant interaction between CS type and Avoidance, *b*_CS type×Avoidance_ = 0.23, SE = 0.074, *p* = 0.003. For the SCRs (Fig. 3d), no effects reached significance (smallest *p* = 0.144).

## Discussion—Experiment 1

Using a novel dimensional measure of US-avoidance, the current experiment replicated some findings. First, the introduction of a competing reward reduced the degree of safety behavior, consistent with studies that measured safety behavior binarily (e.g., Rattel et al., 2017; Volders et al., 2015). This reduction of safety behavior was accompanied by an increase in conditioned fear responses, specifically, a decrease in US-avoidance predicted a significantly stronger increase in both US expectancies and SCRs to the CS+ than the CS–. This pattern aligned with the prediction of the Expectancy model (Lovibond, 2006), suggesting that avoidance modulates conditioned fear responses, mediated by the anticipation of an US. Second, a competing reward incentivized low degree of safety behavior, which in turn initiated extinction learning to the CS+.

As predicted, an increase in US-avoidance on the last trial of Pavlovian extinction predicted a greater increase in expectancy ratings for the CS+ than the CS– on the first trial of test, suggesting a stronger protection from extinction effect with increased US-avoidance in extinction. However, the Reward group did not show a reliable alleviated protection from extinction effect in test when US-avoidance became unavailable. The use of a fixed amount of competing reward may have resulted in different individual evaluations of the reward as being either insufficient or sufficient to motivate low degree of US-avoidance. Therefore, some participants in the Reward group may have excessively engaged in a high degree of US-avoidance, hence reducing the group differences in extinction learning and the subsequent protection from extinction effect.

## Experiment 2

The use of a fixed amount of competing reward in Experiment 1 may not be sensitive enough to examine costly US-avoidance, given potential different individual evaluations of the reward. Therefore, we attempted to adjust the magnitude of the competing reward individually in Experiment 2. Importantly, we matched the level of competing reward to the level of US intensity. That means the adjusted magnitude of competing reward was presumably neither too low that artificially increase the degree of safety behavior nor too high that would artificially induce an opposite pattern. This reward matching procedure was carried out immediately after the US workup procedure; the level of reward was presumed to be merely suffcient to encourage non-avoidance throughout the experiment.

### Method

#### Participants

Psychology undergraduates or residents from Würzburg were recruited and received either course credit or 9€ for participation. A total of 100 participants were recruited, with half of them randomly assigned to the Reward group and the remaining half randomly assigned to the Control group.

#### Apparatus and materials

Apparatus and materials were the same as those used in Experiment 1.

#### Procedure

The procedure was the same as that followed in Experiment 1, except in the following aspect: Immediately after US workup procedure, both Reward and Control groups received a reward-matching procedure. Participants were presented with a series of questions “Are you willing to pay €__ in order to avoid the electrical stimulation?” with the amount of reward ranging from 4 to 30 cents in even numbers (i.e., 4 cents, 6 cents,…28 cents, 30 cents) presented in a randomized order. That means, a total of 14 questions were presented. Participants had to answer either “yes” or “no” to the questions. The amount of competing reward was the amount between the lowest amount that received a “No” and the highest amount that received a “Yes”. For instance, if an individual participant were willing to pay from 4 to 18 cents in favor of avoiding the US (i.e., answering “Yes”) but unwilling to pay from 20 cents onwards (i.e., answering “No”), the amount in between (19 cents) would be used as the maximum competing reward per trial in the following conditioning task. The amount in between was chosen, because it was just before the assumed amount of reward that encourages full disengagement of US-avoidance (cf. Schlund et al., 2016).

#### Results and discussion

The exclusion criterion was identical to Experiment 1. One participant from the Control group did not meet the acquisition criterion and was hence excluded. Two participants (one in each group) were excluded due to excessive movement that might compromise the SCRs. In total, 97 participants (49 in the Reward group, 48 in the Control group) were included for analyses.^2^ Table S2 shows the demographic and DASS-21 data (see Supplementary Materials). No group differences emerged. Similar to Experiment 1, only critical effects involving CS type or Group were reported, while any other effects were reported in the Supplementary Materials.

#### Pavlovian fear acquisition training

**Fig. 4:**
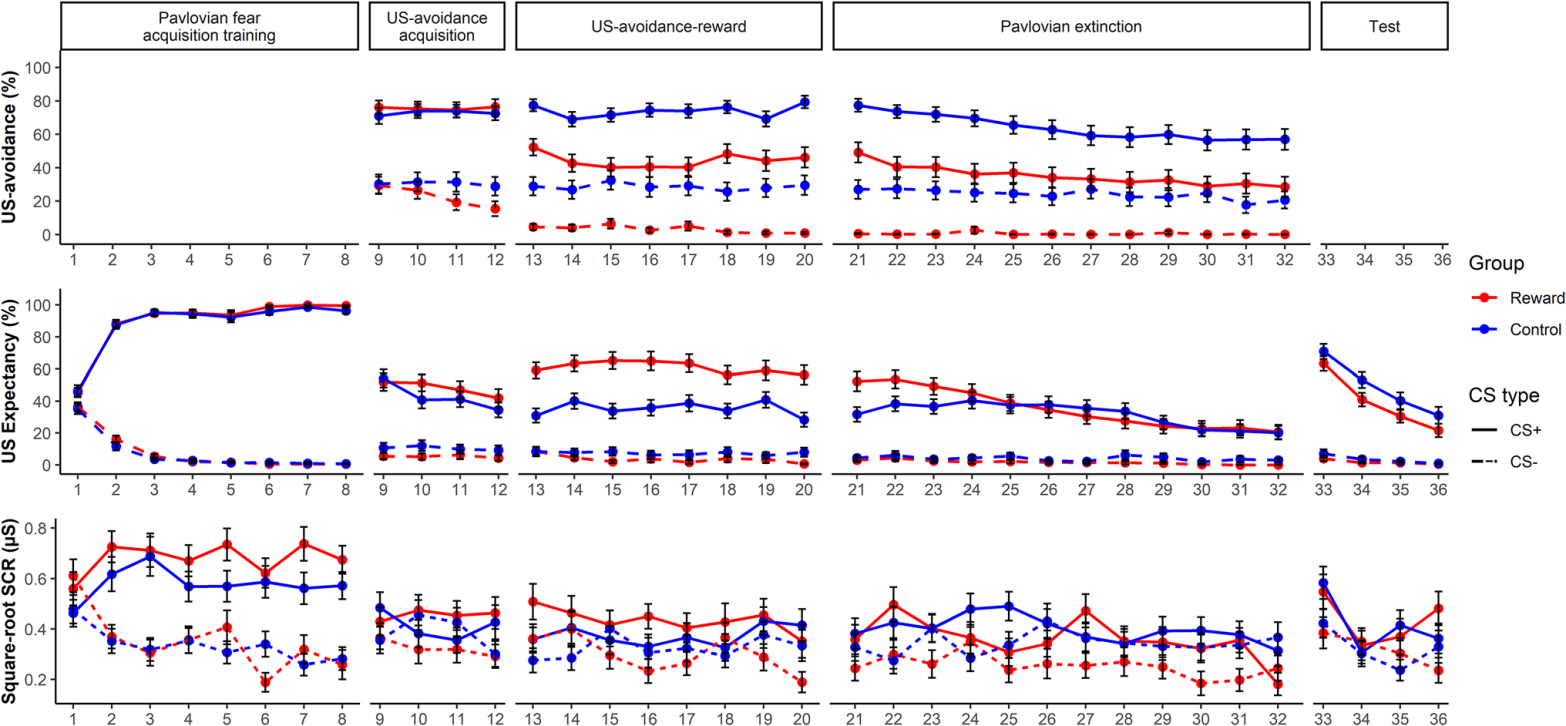
US-avoidance (top panel), US expectancy ratings (middle panel), and square-root SCRs (bottom panel) across all phases between Reward and Control groups in Experiment 2. See the color version of this figure online

Figure 4 shows the US-avoidance, US expectancy ratings and SCRs to the CSs across the different phases. The results of this phase are briefly summarized below (see Supplementary Materials for the detailed analyses). Collectively, both groups acquired differential Pavlovian conditioned fear to the CSs without any group differences in both expectancy ratings and SCRs.

#### Hypothesis 1a: The dynamics between US-avoidance, conditioned fear and competing reward during the acquisition of US-avoidance

##### US-avoidance

Only the interactions are reported here. Compared to the Control group, the Reward group exhibited a larger decrease in US-avoidance to the CS− when transiting from US-avoidance acquisition to US-avoidance-reward, confirmed by a significant interaction between Group and Phase, *b*_Group × Phase_ = − 24.94, SE = 2.16, *p* < 0.001. During the US-avoidance acquisition phase, there was no evidence of group differences in the differential US-avoidance to the CSs, *b*_CS type × Group_ = 0.80, SE = 2.03, *p* = 0.696 (i.e., no group differences in baseline US-avoidance). Importantly, compared to the Control group, the Reward group showed a larger decrease in differential US-avoidance to the CSs in the transition from the US-avoidance acquisition phase to the US-avoidance-reward phase, *b*_CS type×Group×Phase_ = − 14.80, SE = 4.30, *p* < 0.001.

##### Expectancy ratings and SCRs

For the expectancy ratings, the Reward group unexpectedly showed larger differential expectancy ratings to the CSs than the Control group during the US-avoidance acquisition phase, *b*_CS type × Group_ = 23.18, SE = 2.05, *p* < 0.001, despite both groups showed no differences in differential US-avoidance to the CSs in the same phase. The Control group exhibited larger differential ratings to the CSs when transiting from US-avoidance acquisition to US-avoidance-reward, *b*_CS type × Phase_ = 5.33, SE = 2.18, *p* = 0.014. Furthermore, the Reward group showed higher expectancy ratings to the CS− than the Control group in the transition from US-avoidance acquisition to US-avoidance-reward, *b*_Group × Phase_ = 10.84, SE = 2.18, *p* < 0.001. This pattern was due to the Reward group exhibiting higher US-avoidance to the CS− during US-avoidance-reward than the Control group. Importantly, when transiting from the US-avoidance acquisition phase to the US-avoidance-reward phase, the Reward group showed larger differential ratings (higher ratings to the CS+ than the CS−) compared to the Control group, *b*_CS type × Group × Phase_ = 19.03, SE = 4.34, *p* < 0.001.

For the SCRs, the Control group showed stronger responding to the CS+ than the CS− in US-avoidance acquisition, *b*_CS type_ = 0.088, SE = 0.015, *p* < 0.001. Furthermore, the Reward group exhibited larger differential responding to the CSs than the Control group during US-avoidance acquisition, *b*_CS type × Group_ = 0.092, SE = 0.031, *p* = 0.003. No other effects reached significance (smallest *p* = 0.410).

In sum, the introduction of a competing reward decreased US-avoidance, especially to the CS+. Differential expectancy ratings to the CS+ increased presumably due to the decrease in US-avoidance. Similar to Experiment 1, once competing reward was introduced, the Reward group suppressed US-avoidance to the CS− to a greater extent than the Control group.

#### Hypothesis 1b: the dynamics between US-avoidance, competing reward, and extinction learning

##### US-avoidance

On the first extinction trial, the Control group showed stronger US-avoidance to the CS+ than the CS–, supported by a significant main effect of CS type, *b*_CS type_ = 37.33, SE = 0.96, *p* < 0.001. Furthermore, the Control group also exhibited larger differential US-avoidance to the CSs than the Reward group on the first extinction trial, *b*_CS type×Group_ = − 5.20, SE = 1.91, *p* = 0.007. The differential US-avoidance to the CSs decreased across extinction trials in the Control group, *b*_CS type × Trial_ = − 1.39, SE = 0.28, *p* < 0.001. However, similar to Experiment 1, this interaction did not further interact with Group, *b*_CS type×Trial×Group_ = − 0.14, SE = 0.55, *p* = 0.798. This suggests that there was no evidence for any group differences in the decrease in US-avoidance to the CSs across extinction. Consistent with Experiment 1, we observed a significant main effect of Group, *b*_Group_ = − 26.23, SE = 5.08, *p* < 0.001, suggesting that the Reward group showed lower US-avoidance to the CS– on the first extinction trial compared to the Control group. This heightened US-avoidance to the CS− in the Control group remained stable across extinction, *b*_Group×Trial_ = 0.59, SE = 0.39, *p* = 0.132.

##### Expectancy ratings and SCRs

For the expectancy ratings, the Control group showed higher ratings to the CS+ than the CS− on the first extinction trial, *b*_CS type_ = 30.68, SE = 2.18, *p* < 0.001. This differential rating to the CSs on the first extinction trial was larger in the Reward group than the Control group, supported by a significant interaction between CS type and Group, *b*_CS type×Group_ = 5.76, SE = 1.78, *p* = 0.001. Across extinction trials, the Control group exhibited a greater decrease in expectancy ratings to the CS+ than the CS−, b_CS type×Trial_ = -2.2, SE = 0.26, *p* < 0.001. The Reward group also showed a larger decrease in expectancy ratings to the CS– across extinction trials compared to the Control group, *b*_Group×Trial_ = − 0.93, SE = 0.26, *p* < 0.001. Importantly, this decrease in differential ratings for the CSs across extinction was greater in the Reward group than the Control group, *b*_CS type×Trial×Group_ = − 1.51, SE = 0.52, *p* = 0.003. This suggested that extinction learning to the CS+ took place in the Reward group, whereas the Control group showed very limited extinction learning.

The SCRs revealed a relatively irregular pattern. On the first extinction trial, the Control group showed stronger responding to the CS+ than the CS−, supported by a main effect of CS type, *b*_CS type_ = 0.080, SE = 0.015, *p* < 0.001. The Reward group exhibited weaker responding to the CS– on the first extinction trial compared to the Control group, supported by a main effect of Group, *b*_Group_ = − 0.0065, SE = 0.031, *p* = 0.041. No interactions reached significance (smallest *p* = 0.066).

Collectively, the Reward group showed less US-avoidance to the CS+ than the Control group, allowing extinction learning to the CS+ to take place. Extinction learning to the CS+, however, was only observed in the expectancy ratings. Interestingly, the Control group showed an increased degree of US-avoidance to the CS− across extinction.

#### Hypothesis 1c: competing reward reduces protection from extinction

##### Expectancy ratings and SCRs

For the expectancy ratings, the Control group showed higher ratings for the CS+ than the CS− on the last extinction trial, confirmed by a main effect of CS type, *b*_CS type_ = 40.24, SE = 2.56, *p* < 0.001. This differential ratings was greater to the CS+ than the CS− in the transition from the last extinction trial to the first test trial, *b*_CS type×Trial_ = 42.61, SE = 4.46, *p* < 0.001, indicating a protection from extinction effect in the Control group. However, although the Reward group showed a smaller increase in differential ratings to the CSs between Pavlovian extinction and Test when compared to the Control group, this group difference did not reach significance, *b*_CS type×Trial×Group_ = -7.95, SE = 8.91, *p* = 0.373.

The SCRs showed a similar pattern. Responding to the CS+ was greater than the CS− when transiting from Pavlovian extinction to test in the Control group, *b*_CS type × Trial_ = 0.22, SE = 0.073, *p* = 0.003. However, there was no evidence of any group differences in this increase in differential responding to the CSs between the two phases, *b*_CS type×Trial × Group_ = 0.013, SE = 0.15, *p* = 0.931.

In sum, both groups showed an increase in conditioned fear from the last extinction trial to the first test trial, indicating a protection from extinction effect. Surprisingly, no group differences were observed.

#### Hypothesis 2a: a negative linear relationship between US-avoidance and conditioned fear

**Fig. 5:**
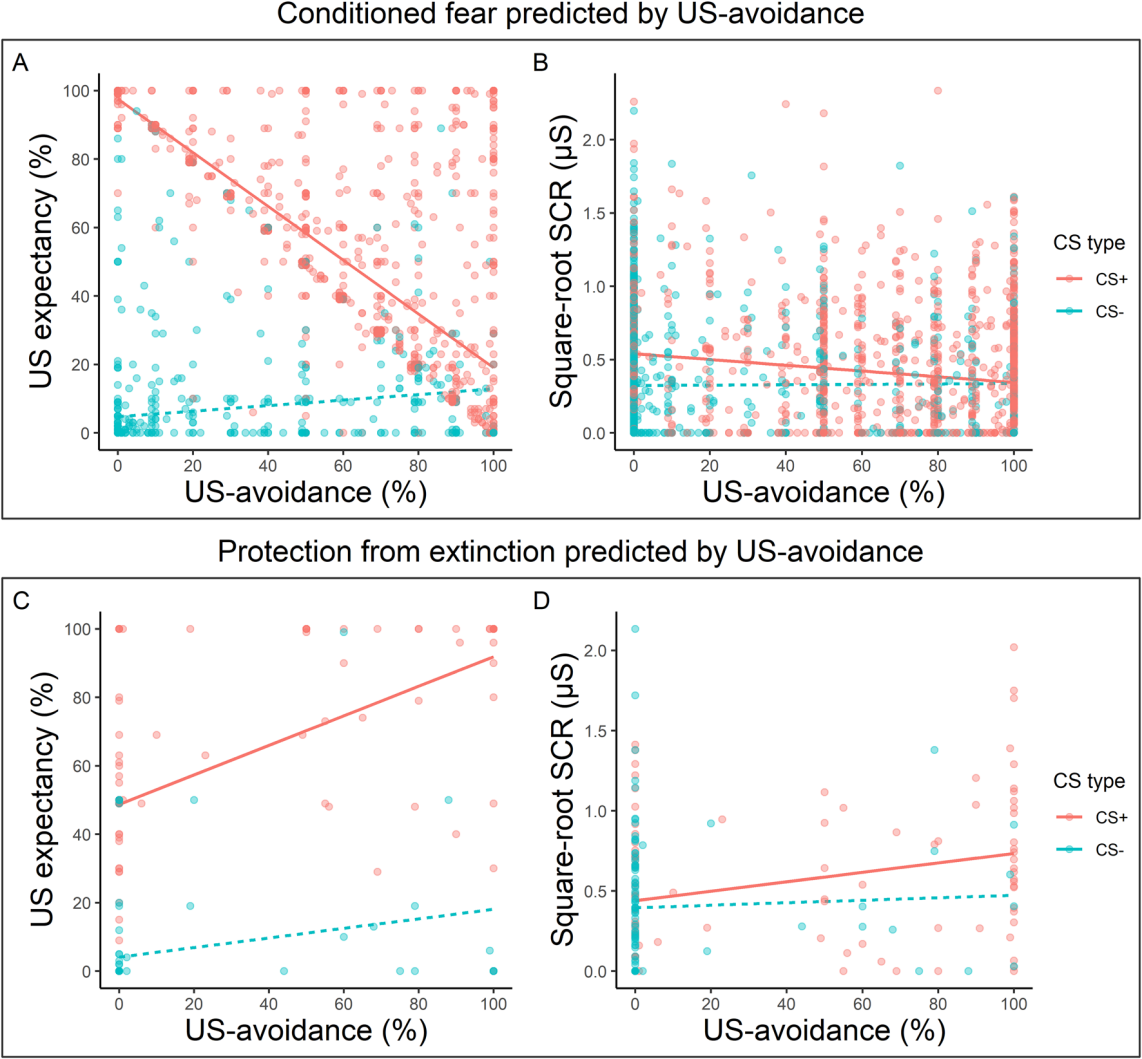
Relationship between US-avoidance and conditioned fear during different phases in Experiment 2. Top Panel. US-avoidance predicts **a** US expectancy ratings and **b** square-root SCRs to the CS+ and the CS− during US-avoidance acquisition and US-avoidance-reward. Bottom Panel. US-avoidance on the last extinction trial predicting **c** US expectancy and **d** square-root SCRs on the first test trial. Red dots represent responding to the CS+ whereas green dots represent responding to the CS−. Darker color indicates more overlapping data points. The lines represent the line of best fit for each CS for visual aid. See the color version of this figure online

Collapsed across groups and both US-avoidance acquisition and US-avoidance-reward, an increase in US-avoidance predicted a more significant decrease in expectancy ratings to the CS+ than the CS− (Fig. 5a), confirmed by a significant interaction between Avoidance and CS type, b_CS type×Avoidance_ = -0.83, SE = 0.025, *p* < 0.001. The SCRs showed a similar pattern (Fig. 5b), in which an increase in US-avoidance led to a greater decrease in responding to the CS+ than the CS−, *b*_CS type×Avoidance_ = − 0.0024, SE = 0.00048, *p* < 0.001.

In sum, an increase in US-avoidance significantly predicted a subsequent decrease in both expectancy ratings and SCRs. This decrease in conditioned fear was greater to the CS+ than the CS−.

#### Hypothesis 2b: A positive linear relationship between US-avoidance and protection from extinction

An increase in US-avoidance on the last extinction trial significantly predicted a stronger increase in expectancy ratings to the CS+ than the CS− on the first test trial, *b*_Avoidance × CS type_ = 0.29, SE = 0.094, *p* = 0.002. However, no effects in the SCR reached significance (smallest *p* = 0.171). This suggested stronger US-avoidance on the last extinction trial predicted a stronger protection from extinction effect, as indicated in the expectancy ratings.

Collectively, the results obtained in Experiment 2 were similar to Experiment 1. Overall, the Reward group displayed less US-avoidance to both CSs, especially to the CS+, which allowed extinction learning to the CS+. However, despite the magnitude of reward was matched to the level of US intensity for each individual participant in the Reward group, which presumably reduced the variability of US-avoidance motivation, we still observed no reliable group differences in protection from extinction. Nonetheless, a dimensional measure of US-avoidance consistently predicted subsequent conditioned fear to the CSs during phases of US-avoidance acquisition, and predicted the magnitude of protection from extinction effect.

## General discussion

Across two experiments using a differential fear and avoidance conditioning procedure, we employed a novel dimensional measure of US-avoidance to examine safety behavior. We expected to replicate findings using a dichotomous measure, in which an introduction of a competing reward would, first, reduce US-avoidance to the CS+, accompanied by an increase in conditioned fear; second, this decrease in US-avoidance would initiate extinction learning to the CS+ when US-administration was discontinued, as indexed by a decrease in US expectancy ratings and SCRs to the CS+; third, would lead to an alleviated protection from extinction effect. Furthermore, we expected that a dimensional measure of US-avoidance to be a sensitive measure to predict subsequent levels of conditioned fear, and that the degree of US-avoidance engagement on the last extinction trial predicted the magnitude of the protection from extinction effect.

As predicted, both experiments showed a decrease in US-avoidance once a competing reward was introduced. This finding is consistent with studies that showed a competing reward acting as an incentive for non-avoidance (e.g., Claes et al., 2014; Pittig, 2019; Rattel et al., 2017; van Damme et al., 2012). Furthermore, the low degree of US-avoidance to the CS+ in the Reward group was accompanied by a high level of US expectancy ratings to the CS+ during the US-avoidance-reward phase. This means that a competing reward encouraged participants to tolerate the US in favor for the reward (i.e., fear-opposite action; Pittig, 2019; Pittig & Dehler, 2019). Second, across both experiments, the Reward group continued to show lower degree of US-avoidance to the CS+ than the Control group throughout the Pavlovian extinction phase. This led to a high level of US expectancies to the CS+ on early extinction trials, which, however, gradually decreased, indicating extinction learning. This suggested that a low degree of US-avoidance induced by a competing reward enabled extinction learning. In contrast, the Control group continued to show a high degree of US-avoidance to the CS+ throughout Pavlovian extinction, accompanied by low levels of US expectancies, suggesting that participants attributed US omission to their engagement in US-avoidance. This means the similarly low levels of US expectancy between groups at the end of the Pavlovian extinction phase were attributed to different causes: In the Reward group, this was mainly attributed to extinction learning to the CS+ , whereas in the Control group, this was mainly attributed to the high degree of US-avoidance. In sum, this finding aligned with studies that showed excessive engagement in safety behavior preventing extinction learning (Lovibond et al., 2009; Pittig, 2019; Rattel et al., 2017).

Contrary to our third prediction, both groups showed a similar level of protection from extinction effect in test. We hypothesized that the fixed amount of competing reward in Experiment 1 may be sufficient to encourage low degree of avoidance to some participants while trivial to some participants, therefore decreasing the effect of competing reward on US-avoidance. In light of this, we matched the magnitude of reward to the US intensity for each individual participant in the Reward group in Experiment 2. However, despite our anticipation that this matching procedure would have increased the sensitivity to detect a reduced protection from extinction effect in the Reward group, we still observed no group differences in protection from extinction.

The current results also demonstrated a dimensional measure of US-avoidance sufficiently predicted subsequent levels of conditioned fear and the magnitude of protection from extinction effect. First, an increase in US-avoidance predicted a stronger decrease in US expectancies and SCRs to the CS+ when compared to the CS− during the phases of US-avoidance acquisition. This pattern aligned with the notion of the Expectancy model that avoidance modulates subsequent conditioned fear responses to the CS+ via a process of mental anticipation of an aversive outcome. This means, when a high degree of US-avoidance to the CS+ was made, the chance of US delivery was anticipated to a lesser extent, leading to low levels of conditioned fear to the CS+ (see Mitchell, Lovibond & De Houwer, 2009). In contrast, US-avoidance to the CS− showed little predictiveness to the subsequent conditioned fear, suggesting that participants knew that the CS– would not be followed by an aversive outcome regardless of US-avoidance.

Despite the null group difference in protection from extinction, we found that an increase in US-avoidance on the last extinction trial significantly predicted stronger conditioned fear to the CS+ than the CS− on the first test trial. This finding suggests that an individual engaging in US-avoidance to a greater extent would show a stronger protection from extinction effect. This is in line with the notion of the Expectancy model, suggesting that the more likely one engage in US-avoidance, the more likely one would attribute the absence of an aversive outcome to the avoidance response, despite the aversive outcome no longer follows the CS+ regardless of avoidance responses. Notably, the dimensional measure of US-avoidance allowed US-avoidance on a single trial to predict the magnitude of protection from extinction.

The unexpected null group difference in protection from extinction were presumably driven by other potential factors. One potential candidate is insufficient expectancy violation. According to the inhibitory learning model of extinction (Craske, Treanor, Conway, Zbozinek, & Vervliet, 2014), treatment outcome of exposure-based therapies depends on to what extent a patient’s threat belief has been challenged throughout therapy sessions (Pittig et al., submitted). That is, a mismatch between threat expectancy and the actual outcome. The larger this violation of expectancy, the more extinction learning takes place and therefore the better the treatment outcome. However, a dimensional measure of US-avoidance allowed participants to not fully disengage from US-avoidance during extinction. This means, violation of US expectancy could be partially attributed to genuine extinction learning and partially attributed to US-avoidance. Therefore, expectancy violation was not maximized during extinction, decreasing any observable reductions in the subsequent protection from extinction effect. Another potential factor in play was the change in context between the Pavlovian extinction and test phase. Extinction learning is vulnerable to a change in the spatio-temporal context, that is, an extinguished fear-related stimulus presented in a context different from the extinction context would trigger a return of fear (Bouton, 2002; Bouton & Bolles, 1979; Bouton & King, 1983). In the current study, US-avoidance was available during Pavlovian extinction, but was removed during test. The removal of US-avoidance might have represented a contextual change, resulting in an increase of conditioned fear to the CS+. This increase of conditioned fear might have reduced any observable group differences in protection from extinction. In support, studies showed that the removal of US-avoidance availability from extinction to test resulted in a return of fear (Vervliet & Indeuku, 2015, Pittig, 2019; Rattel et al., 2017, but see Lovibond et al., 2009). A final potential factor in play was the use of a strategy. Some participants in the Reward group might have used a strategy to gain the competing reward via non-avoidance to the CS− only. This means that participants could still obtain monetary gain by not avoiding a safety cue, rendering avoidance of CS+ relatively less costly. This strategy might have artificially increased US-avoidance to the CS+ in the Reward group, reduced extinction learning to the CS+ and therefore potentially contributed to the null group difference in protection from extinction. Future studies may reduce the use of this strategy by reducing the amount of CS– trials or employing a single-cue conditioning paradigm, which the latter has been successfully employed in human fear conditioning (e.g., Baas, 2013; Lee, Hayes, & Lovibond, 2018; Pittig, 2019; Wong & Lovibond, 2017).

Both experiments consistently revealed a novel finding regarding the link between low-cost avoidance and US expectancies to a safety cue. The Control group showed higher degree of US-avoidance to the CS– than the Reward group in both US-avoidance-reward and Pavlovian extinction phases. Importantly, this group difference was not due to the Control group exhibiting stronger conditioned fear to the CS– nor a stronger tendency to generally engage with higher degree of US-avoidance (see Supplementary Materials). This strongly suggests that low-cost US-avoidance does not fully reflect the fear-related component, thereby not accurately reflecting how safety behavior is motivated by fear. In fact, the minimal cost of US-avoidance potentially encouraged participants to engage in a higher degree of US-avoidance to the CS– despite a low level of conditioned fear to it. This discrepancy between conditioned fear and low-cost safety behavior is sometimes referred to as a ‘better safe than sorry’ strategy (e.g., Lommen et al., 2010). However, a ‘better safe than sorry’ strategy implies that participants engage in safety behavior due to the uncertainty of US occurrence. In the current study, participants showed a steady, low level of US expectancies to the CS- prior to US-avoidance acquisition, suggesting a certain absence of an US. Therefore, a ‘why not’ strategy may more suitably describe the discrepancy between conditioned fear and low-cost safety behavior in the current study (i.e., I know the CS– is safe but avoidance response costs nothing, so why not avoid?).

This novel finding has important methodological implications. We should cautiously interpret whether low-cost avoidance is fully motivated by conditioned fear (cf. Krypotos, Effting, Kindt & Beckers, 2015). Specifically, some studies found a high degree of low-cost avoidance to the CS+ after response prevention extinction (e.g., Krypotos & Engelhard, 2019; Vervliet & Indekeu, 2015; but see Krypotos & Engelhard, 2018), presumably caused by a return of Pavlovian fear. However, the current findings favor an alternative interpretation that the heightened avoidance was encouraged by the minimal cost to perform it rather than reflecting a return of fear. In support to this interpretation, incorporating a cost to safety behavior led to little to no avoidance responses to the CS+ after response prevention extinction (Vervliet, Lange & Milad, 2017).

The group differences in US-avoidance to the CS+ and CS− suggested that different processes may modulate the link between conditioned fear and US-avoidance. For instance, the lower degree of US-avoidance to the CS+ in the Reward group reflected a fear-opposite action (e.g., Pittig, 2019; Pittig & Dehler, 2019) modulated by the opportunity cost of safety behavior. In contrast, the elevated US-avoidance to the CS– in the Control group reflected a discrepancy between conditioned fear and US-avoidance (e.g., “why not” strategy) due to the minimal cost of US-avoidance. These findings suggested that a dimensional measure of US-avoidance was sensitive to reveal these different processes linked to safety behavior, providing a promising method for detecting other contributing factors to the acquisition of safety behavior.

Two findings in the current study have important implications in a clinical context. First, the null group difference in protection from extinction was presumably due to expectancy violation not being maximized, given that participants could still engage in safety behavior to a small extent during extinction. This speculation suggests that even partial engagement in safety behavior during exposure sessions is sufficient for one to attribute the absence of an aversive outcome to safety behavior, thus impeding treatment outcome. Second, the Control group showed stronger safety behavior to the safety cue compared to the Reward group, presumably due to the low-cost of safety behavior. This group difference in US-avoidance to the CS– was presumably due to an excessive engagement in low-cost safety behavior (e.g., “why not” strategy). This calls for the attention towards preventing unnecessary low-cost safety behavior to safety cues or situations in individuals with anxiety-related disorders. Past studies showed that clinical samples tended to infer the presence of potential threat via their safety behavior (Gangemi, Mancini, & van den Hout, 2012; van den Hout et al., 2014; Engelhard, van Uijen, van Seters, & Velu, 2015). Combined with the current findings, this suggests that excessive engagement in low-cost behavior in individuals with anxiety-related disorders may paradoxically increase their fear to other safety cues or situations. Collectively, the current study suggests completely preventing any engagement in safety behavior, if possible, to maximize treatment outcome (see also Blakey & Abramowitz, 2016).

The current study does have some limitations. First, we found only a few significant effects on the skin conductance measure. Importantly, this was not due to a failure of fear acquisition in skin conductance, given that participants developed discriminative skin conductance responding to the CSs in the Pavlovian fear acquisition training phase in both experiments. One reason would be habituation of skin conductance responses to the CSs and the US (Sokolov, 1960). Given the large amount of trials in the current study, habituation of skin conductance may have minimized any expected effects, especially to trials presented late in the experiment. Second, this study did not directly compare a dimensional measure of US-avoidance to a binary measure. Therefore, it remained unclear whether a dimensional measure of US-avoidance fares any better than a binary measure of avoidance. Nonetheless, we see a dimensional measure of US-avoidance as an assessment of the extent of safety behavior engagement. In addition, the continuous nature of this measure allows it to more sensitively delineate the relationship between safety behavior and conditioned fear. Third, the CSs were not counterbalanced across participants, potentially confounding the results. Forth, the questions in the reward matching procedure in Experiment 2 were presented with negative connotation. Some participants might have misinterpreted the question as in they had to pay out of their own pockets. Therefore, they might only answered “yes” to the lower range of monetary value despite the real level of individually matched competing reward might have been higher than that. This might have led to a considerable amount of participants in the Reward group engaging in a high degree of US-avoidance, hence reducing the effect of individually matched competing reward on protection from extinction. Future studies can phrase the reward matching questions in a positive connotation, for instance, “Are you willing to tolerate an electrical stimulation if you are given 0.10€?”.

In conclusion, the current study developed a dimensional measure of US-avoidance that had a negative linear relationship with US administration and the amount of competing reward. The introduction of a competing reward reduced the degree of US-avoidance and initiated extinction learning to the CS+. However, the competing reward had no apparent effect on the alleviation of protection from extinction. The apparent null group difference in protection from extinction may be attributed to the competing reward not strong enough to motivate fear-opposite action (especially in Experiment 1), expectancy violation not being maximized, a change in context from Pavlovian extinction to test, a strategy to not avoid the CS– only, or a combination of these factors. A novel finding was the elevated US-avoidance to the CS– in the Control group in both experiments due to the minimal cost of US-avoidance. This finding further confirmed that low-cost avoidance does not fully reflect the fear-related component (i.e., avoidance is not only motivated by fear to the feared stimulus). Furthermore, combined with past findings (Gangemi et al., 2012; van den Hout et al., 2014), this novel pattern suggested that safety behavior with minimal cost may paradoxically increase fear to safety cues in individuals with anxiety-related disorders, therefore clinicians should attend to low-cost safety behavior that may have gone unnoticed.

## Supplementary Information

Below is the link to the electronic supplementary material.

Supplementary file1 (DOCX 87 KB)

## Data Availability

The datasets generated and analysed during the current study are available in the Open Science Framework repository, https://osf.io/z7jpt/.

## Footnotes

1 Results remained similar with all participants included (see Supplementary Materials).

2 Results remained similar with all participants included.
