# Supplementary material for: A dimensional measure of safety behavior: A non-dichotomous assessment of costly avoidance in human fear conditioning
Source: Psychol Res. 2021 Mar 4;86(1):312–30. doi: 10.1007/s00426-021-01490-w (PMC8821075; doi:10.1007/s00426-021-01490-w)
Supplement: Supplementary file 1 — Supplementary file1 (DOCX 87 KB) [file 426_2021_1490_MOESM1_ESM.docx]

Supplementary Material

Experiment 1

Table S1.
Demographic and DASS-21 data

|  | Reward group (n=49) | Control group (n=49) | F or χ^2^ | *p* |
| --- | --- | --- | --- | --- |
| Age | 22.8 (3.6) | 23.3(4.4) | 0.4 | 0.5 |
| Sex - Females | 40(82%) | 42 (86%) | 0.3 | 0.6 |
| US intensity | 0.8mA (0.7) | 0.7mA (0.4) | 2.2 | 0.1 |
| DASS 21-Anxiety | 4.1 (4.0) | 4.7 (4.8) | 0.5 | 0.5 |
| DASS 21-Depression | 5.3 (5.7) | 7.5 (7.8) | 2.5 | 0.1 |
| DASS 21-Stress | 8.2 (6.7) | 10.7 (7.9) | 2.8 | 0.1 |

Note. Means (and deviations)

Experiment 1(Hypotheses 1a & 1b):

US-avoidance

| Main effects and interactions | Pavlovian fear acquisition training | | | US-avoidance acquisition | | US-avoidance-reward | | Pavlovian extinction | | | Test | | |
| --- | --- | --- | --- | --- | --- | --- | --- | --- | --- | --- | --- | --- | --- |
|  | b | SE | *p* | b | SE | | *p* | b | SE | *p* | b | SE | *p* |
| CS type | NA | | | 37.81 | 1.10 | | <.001 | 33.80 | 0.94 | <.001 | NA | | |
| Phase/Trial |  |  |  | -18.34 | 1.16 | | <.001 | -1.02 | 0.14 | <.001 |  |  |  |
| Group |  |  |  | -22.70 | 4.32 | | <.001 | -30.92 | 5.46 | <.001 |  |  |  |
| CS type*Phase/Trial |  |  |  | 5.40 | 2.20 | | .014 | -1.90 | 0.27 | <.001 |  |  |  |
| CS type*Group |  |  |  | -1.53 | 2.07 | | .462 | -2.14 | 1.87 | .252 |  |  |  |
| Group*Phase/Trial |  |  |  | -35.95 | 2.20 | | <.001 | 0.015 | 0.27 | .957 |  |  |  |
| CS type*Phase/Trial*Group |  |  |  | -10.23 | 4.39 | | .020 | 0.73 | 0.54 | .177 |  |  |  |

Experiment 1 (Hypotheses 1a & 1b):

US-expectancy

| Main effects and interactions | Pavlovian fear acquisition training | | | | | US-avoidance acquisition | | US-avoidance-reward | | Pavlovian extinction | | | Test | | | | |
| --- | --- | --- | --- | --- | --- | --- | --- | --- | --- | --- | --- | --- | --- | --- | --- | --- | --- |
|  | b | | SE | | *p* | b | SE | | *p* | b | SE | *p* | b | SE | | | *p* |
| CS type | 81.26 | 1.09 | | <.001 | | 30.74 | 1.04 | | <.001 | 24.73 | 0.84 | <.001 | 40.33 | | 1.46 | <.001 | |
| Phase/Trial | 0.80 | 0.21 | | <.001 | | 1.09 | 1.10 | | .323 | -0.96 | 0.12 | <.001 | -6.96 | | 0.65 | <.001 | |
| Group | -0.27 | 0.95 | | .775 | | 12.95 | 3.04 | | <.001 | 8.03 | 3.17 | .013 | -4.56 | | 2.96 | .126 | |
| CS type*Phase/Trial | 9.30 | 0.34 | | <.001 | | 5.15 | 2.11 | | .015 | -1.65 | 0.24 | <.001 | -10.93 | | 1.23 | <.001 | |
| CS type*Group | -0.36 | 1.54 | | .816 | | 14.96 | 1.99 | | <.001 | 13.68 | 1.63 | <.001 | -4.99 | | 2.75 | .070 | |
| Group*Phase/Trial | -0.021 | 0.34 | | .949 | | 24.60 | 2.11 | | <.001 | -1.54 | 0.24 | <.001 | -0.42 | | 1.23 | .732 | |
| CS type*Phase/Trial*Group | -0.23 | 0.67 | | .734 | | 32.20 | 4.17 | | <.001 | -2.34 | 0.47 | <.001 | -1.64 | | 2.46 | .504 | |

Experiment 1 (Hypotheses 1a & 1b):

SCR

| Main effects and interactions | Pavlovian fear acquisition training | | | | | US-avoidance acquisition | | US-avoidance-reward | | Pavlovian extinction | | | Test | | | | |
| --- | --- | --- | --- | --- | --- | --- | --- | --- | --- | --- | --- | --- | --- | --- | --- | --- | --- |
|  | b | | SE | | *p* | b | SE | | *p* | b | SE | *p* | b | SE | | | *p* |
| CS type | 0.20 | 0.016 | | <.001 | | 0.080 | 0.014 | | <.001 | 0.085 | 0.016 | <.001 | 0.089 | | 0.026 | <.001 | |
| Phase/Trial | -0.0053 | 0.0035 | | .128 | | -0.019 | 0.015 | | .205 | -0.0012 | 0.0023 | .598 | -0.024 | | 0.012 | .039 | |
| Group | -0.064 | 0.051 | | .210 | | -0.060 | 0.048 | | 0.217 | -0.10 | 0.052 | .054 | -0.099 | | 0.055 | .076 | |
| CS type*Phase/Trial | 0.025 | 0.0070 | | <.001 | | 0.078 | 0.030 | | .001 | -0.0050 | 0.0046 | .277 | -0.034 | | 0.023 | .138 | |
| CS type*Group | -8.0*10^-4^ | 0.032 | | .980 | | 0.040 | 2.84 | | .163 | 0.018 | 0.032 | .579 | -0.071 | | 0.052 | .169 | |
| Group*Phase/Trial | -0.0063 | 0.0070 | | .367 | | 0.024 | 0.030 | | .431 | 0.0030 | 0.0046 | .520 | 0.025 | | 0.023 | .286 | |
| CS type*Phase/Trial*Group | 0.012 | 0.014 | | .402 | | 0.061 | 0.060 | | .314 | -0.00062 | 0.0082 | .947 | 3.5*10^-5^ | | 0.046 | .999 | |

Experiment 1 – Additional models (Hypotheses 1c, 2a & 2b):

Expectancy ratings

| Main effects and interactions | Anticipatory fear predicted by US-avoidance during phases of US-avoidance acquisition | | | Protection from extinction | | | Protection from extinction predicted by US-avoidance in Extinction | | | | |
| --- | --- | --- | --- | --- | --- | --- | --- | --- | --- | --- | --- |
|  | b | SE | *p* | b | SE | *p* | b | SE | | | *p* |
| CS type | 41.59 | 1.18 | <.001 | 36.63 | 2.56 | <.001 | 53.98 | | 3.12 | <.001 | |
| US-avoidance | -0.29 | 0.017 | <.001 | NA | | | 0.24 | | 0.040 | <.001 | |
| CS type*US-avoidance | -0.70 | 0.026 | <.001 |  |  |  | 0.23 | | 0.074 | .003 | |
| Phase/Trial | NA | | | 24.19 | 2.56 | <.001 | NA | | | | |
| Group |  |  |  | 0.18 | 2.71 | .948 |  |  |  |  |  |
| CS type*Phase/Trial |  |  |  | 45.05 | 4.37 | <.001 |  |  |  |  |  |
| CS type*Group |  |  |  | 2.95 | 4.37 | .500 |  |  |  |  |  |
| Group*Phase/Trial |  |  |  | -6.50 | 4.37 | .138 |  |  |  |  |  |
| CS type*Phase/Trial*Group |  |  |  | -10.84 | 8.72 | .215 |  |  |  |  |  |

Experiment 1 – (Hypotheses 1c, 2a & 2b):

SCRs

| Main effects and interactions | Anticipatory fear predicted by US-avoidance during phases of US-avoidance acquisition | | | Protection from extinction | | | Protection from extinction predicted by US-avoidance in Extinction | | | |
| --- | --- | --- | --- | --- | --- | --- | --- | --- | --- | --- |
|  | b | SE | *p* | b | SE | *p* | b | SE | | *p* |
| CS type | 0.091 | 0.017 | <.001 | 0.10 | 0.044 | .020 | 0.11 | | 0.054 | .047 |
| US-avoidance | -0.00031 | 0.00025 | .214 | NA | | | 0.0014 | | 0.00081 | .098 |
| CS type*US-avoidance | -0.0015 | 0.00043 | <.001 |  |  |  | 8.4*10^-5^ | | 0.0013 | .950 |
| Phase/Trial | NA | | | 0.028 | 0.044 | .520 | NA | | | |
| Group |  |  |  | -0.11 | 0.061 | .081 |  |  |  |  |
| CS type*Phase/Trial |  |  |  | 0.071 | 0.088 | .423 |  |  |  |  |
| CS type*Group |  |  |  | -0.011 | 0.088 | .905 |  |  |  |  |
| Group*Phase/Trial |  |  |  | -0.076 | 0.088 | .389 |  |  |  |  |
| CS type*Phase/Trial*Group |  |  |  | 0.057 | 0.18 | .747 |  |  |  |  |

Experiment 2:

|  | Reward group (n=49) | Control group (n=48) | F or χ^2^ | *p* |
| --- | --- | --- | --- | --- |
| Age | 23.2 (3.0) | 24.3 (3.8) | 1.7 | 0.2 |
| Sex - Females | 37 (76%) | 29 (60%) | 2.9 | 0.09 |
| US intensity | 1.4mA (0.7) | 1.6mA (1.2) | 0.9 | 0.3 |
| DASS 21-Anxiety | 2.6 (2.4) | 3.8 (4.4) | 2.8 | 0.1 |
| DASS 21-Depression | 5.0 (4.6) | 4.8 (5.8) | 0.05 | 0.8 |
| DASS 21-Stress | 8.7 (7.2) | 8.0 (6.8) | 0.3 | 0.6 |

Table S2.
Demographic and DASS-21 data

Note. Means (and standard deviations)

Experiment 2 (Hypotheses 1a & 1b):

US-avoidance

| Main effects and interactions | Pavlovian fear acquisition training | | | US-avoidance acquisition | | US-avoidance-reward | | Pavlovian extinction | | | Test | | |
| --- | --- | --- | --- | --- | --- | --- | --- | --- | --- | --- | --- | --- | --- |
|  | β | SE | *p* | β | SE | | *p* | β | SE | *p* | β | SE | *p* |
| CS type | NA | | | 44.68 | 1.05 | | <.001 | 37.33 | 0.96 | <.001 | NA | | |
| Phase/Trial |  |  |  | -13.01 | 1.11 | | <.001 | -1.03 | 0.14 | <.001 |  |  |  |
| Group |  |  |  | -19.18 | 4.40 | | <.001 | -26.23 | 5.08 | <.001 |  |  |  |
| CS type*Phase/Trial |  |  |  | -4.54 | 2.16 | | .035 | -1.39 | 0.28 | <.001 |  |  |  |
| CS type*Group |  |  |  | 0.80 | 2.03 | | .696 | -5.2- | 1.01 | .007 |  |  |  |
| Group*Phase/Trial |  |  |  | -24.94 | 2.16 | | <.001 | 0.52 | 0.28 | .062 |  |  |  |
| CS type*Phase/Trial*Group |  |  |  | -14.80 | 4.30 | | <.001 | -0.14 | 0.55 | .798 |  |  |  |

Experiment 2 (Hypotheses 1a & 1b):

US-expectancy

| Main effects and interactions | Pavlovian fear acquisition training | | | | | US-avoidance acquisition | | US-avoidance-reward | | Pavlovian extinction | | | Test | | | | |
| --- | --- | --- | --- | --- | --- | --- | --- | --- | --- | --- | --- | --- | --- | --- | --- | --- | --- |
|  | β | | SE | | *p* | β | SE | | *p* | β | SE | *p* | β | SE | | | *p* |
| CS type | 81.35 | 0.99 | | <.001 | | 41.00 | 1.06 | | <.001 | 30.68 | 2.18 | <.001 | 41.29 | | 1.53 | <.001 | |
| Phase/Trial | 0.62 | 0.22 | | .004 | | 0.27 | 1.13 | | .809 | -1.36 | 0.13 | <.001 | -5.68 | | 3.00 | .061 | |
| Group | 0.90 | 0.99 | | .366 | | 7.34 | 3.06 | | .019 | 0.54 | 2.82 | .848 | -7.46 | | 0.68 | <.001 | |
| CS type*Phase/Trial | 8.9 | 0.37 | | <.001 | | 5.33 | 2.18 | | .014 | -2.23 | 0.26 | <.001 | -11.88 | | 1.28 | <.001 | |
| CS type*Group | 0.31 | 1.68 | | .854 | | 23.18 | 2.05 | | <.001 | 5.76 | 1.78 | .001 | -8.17 | | 2.86 | .004 | |
| Group*Phase/Trial | 0.0013 | 0.37 | | .997 | | 10.84 | 2.18 | | <.001 | -0.93 | 0.26 | <.001 | 0.30 | | 1.28 | .814 | |
| CS type*Phase/Trial*Group | 1.01 | 0.73 | | .168 | | 19.03 | 4.34 | | <.001 | -1.51 | 0.52 | .003 | -1.22 | | 2.56 | .633 | |

Experiment 2 (Hypotheses 1a & 1b):

SCRs

| Main effects and interactions | Pavlovian fear acquisition training | | | | | US-avoidance acquisition | | US-avoidance-reward | | Pavlovian extinction | | | Test | | | | |
| --- | --- | --- | --- | --- | --- | --- | --- | --- | --- | --- | --- | --- | --- | --- | --- | --- | --- |
|  | β | | SE | | *p* | β | SE | | *p* | β | SE | *p* | β | SE | | | *p* |
| CS type | 0.29 | 0.018 | | <.001 | | 0.088 | 0.015 | | <.001 | 0.080 | 0.015 | <.001 | 0.10 | | 0.026 | <.001 | |
| Phase/Trial | -0.012 | 0.0039 | | .002 | | -0.036 | 0.016 | | .029 | -0.0061 | 0.0022 | .005 | -0.039 | | 0.011 | <.001 | |
| Group | 0.059 | 0.043 | | .178 | | 0.0091 | 0.033 | | .785 | -0.0065 | 0.031 | .041 | 0.0062 | | 0.048 | .897 | |
| CS type*Phase/Trial | 0.034 | 0.0078 | | <.001 | | 0.012 | 0.033 | | .706 | -0.0080 | 0.0044 | .066 | 0.0063 | | 0.023 | .787 | |
| CS type*Group | 0.086 | 0.036 | | .017 | | 0.092 | 0.031 | | .003 | 0.049 | 0.030 | .104 | 0.021 | | 0.052 | .686 | |
| Group*Phase/Trial | -0.0043 | 0.0078 | | .581 | | 0.027 | 0.033 | | .410 | -0.0069 | 0.0044 | .115 | 0.012 | | 0.023 | .599 | |
| CS type*Phase/Trial*Group | 0.020 | 0.016 | | .206 | | -0.018 | 0.065 | | .776 | 0.0037 | 0.0087 | .674 | 0.054 | | 0.047 | .245 | |

Experiment 2 – Additional models (Hypotheses 1c, 2a & 2b):

Expectancy ratings

| Main effects and interactions | Anticipatory fear predicted by US-avoidance during phases of US-avoidance acquisition | | | Protection from extinction | | | Protection from extinction predicted by US-avoidance in Extinction | | | | |
| --- | --- | --- | --- | --- | --- | --- | --- | --- | --- | --- | --- |
|  | β | SE | *p* | β | SE | *p* | β | SE | | | *p* |
| CS type | 59.55 | 1.25 | <.001 | 40.24 | 2.56 | <.001 | 50.07 | | 3.35 | <.001 | |
| US-avoidance | -0.42 | 0.018 | <.001 | NA | | | 0.35 | | 0.055 | <.001 | |
| CS type*US-avoidance | -0.83 | 0.025 | <.001 |  |  |  | 0.29 | | 0.094 | .002 | |
| Phase/Trial | NA | | | 25.34 | 2.56 | <.001 | NA | | | | |
| Group |  |  |  | -3.23 | 2.82 | .254 |  |  |  |  |  |
| CS type*Phase/Trial |  |  |  | 42.61 | 4.46 | <.001 |  |  |  |  |  |
| CS type*Group |  |  |  | -0.55 | 4.46 | .902 |  |  |  |  |  |
| Group*Phase/Trial |  |  |  | -4.10 | 4.46 | .359 |  |  |  |  |  |
| CS type*Phase/Trial*Group |  |  |  | -7.95 | 8.91 | .373 |  |  |  |  |  |

Experiment 2 – Additional models (Hypotheses 1c, 2a & 2b):

SCRs

| Main effects and interactions | Anticipatory fear predicted by US-avoidance during phases of US-avoidance acquisition | | | Protection from extinction | | | Protection from extinction predicted by US-avoidance in Extinction | | | |
| --- | --- | --- | --- | --- | --- | --- | --- | --- | --- | --- |
|  | β | SE | *p* | β | SE | *p* | β | SE | | *p* |
| CS type | 0.14 | 0.020 | <.001 | 0.052 | 0.037 | .167 | 0.074 | | 0.049 | .133 |
| US-avoidance | -0.0012 | 0.00028 | <.001 | NA | | | 0.0027 | | 0.00078 | <.001 |
| CS type*US-avoidance | -0.0024 | 0.00048 | <.001 |  |  |  | ^0.0010^ | | 0.0015 | .493 |
| Phase/Trial | NA | | | 0.21 | 0.037 | <.001 | NA | | | |
| Group |  |  |  | -0.082 | 0.053 | .122 |  |  |  |  |
| CS type*Phase/Trial |  |  |  | 0.22 | 0.073 | .003 |  |  |  |  |
| CS type*Group |  |  |  | -0.0041 | 0.073 | .056 |  |  |  |  |
| Group*Phase/Trial |  |  |  | 0.090 | 0.073 | .221 |  |  |  |  |
| CS type*Phase/Trial*Group |  |  |  | 0.013 | 0.15 | .931 |  |  |  |  |

Experiment 1 with all participants included (Hypotheses 1a & 1b):

US-avoidance

| Main effects and interactions | Pavlovian fear acquisition training | | | US-avoidance acquisition | | US-avoidance-reward | | Pavlovian extinction | | | Test | | |
| --- | --- | --- | --- | --- | --- | --- | --- | --- | --- | --- | --- | --- | --- |
|  | β | SE | *p* | β | SE | | *p* | β | SE | *p* | β | SE | *p* |
| CS type | NA | | | 38.39 | 3.16 | | <.001 | 34.14 | 0.94 | <.001 | NA | | |
| Phase/Trial |  |  |  | -18.37 | 1.15 | | <.001 | -1.00 | 0.14 | <.001 |  |  |  |
| Group |  |  |  | -22.81 | 4.26 | | <.001 | -31.29 | 5.38 | <.001 |  |  |  |
| CS type*Phase/Trial |  |  |  | 4.79 | 2.18 | | .028 | -1.87 | 0.27 | <.001 |  |  |  |
| CS type*Group |  |  |  | -2.82 | 2.05 | | .170 | -4.06 | 1.85 | .029 |  |  |  |
| Group*Phase/Trial |  |  |  | -36.03 | 2.18 | | <.001 | 0.0066 | 0.27 | .980 |  |  |  |
| CS type*Phase/Trial*Group |  |  |  | -11.03 | 4.35 | | .011 | 0.70 | 0.54 | .193 |  |  |  |

Experiment 1 with all participants included (Hypotheses 1a & 1b):

US-expectancy

| Main effects and interactions | Pavlovian fear acquisition training | | | | | US-avoidance acquisition | | US-avoidance-reward | | Pavlovian extinction | | | Test | | | | |
| --- | --- | --- | --- | --- | --- | --- | --- | --- | --- | --- | --- | --- | --- | --- | --- | --- | --- |
|  | β | | SE | | *p* | β | SE | | *p* | β | SE | *p* | β | SE | | | *p* |
| CS type | 80.21 | 0.97 | | <.001 | | 30.72 | 2.30 | | <.001 | 24.72 | 2.34 | <.001 | 39.98 | | 1.44 | <.001 | |
| Phase/Trial | 0.70 | 0.21 | | <.001 | | 0.84 | 1.09 | | .443 | -0.97 | 0.12 | <.001 | -6.99 | | 0.65 | <.001 | |
| Group | 0.32 | 1.03 | | .755 | | 13.50 | 3.00 | | <.001 | 8.33 | 3.12 | .009 | -4.06 | | 2.94 | .171 | |
| CS type*Phase/Trial | 9.11 | 0.35 | | <.001 | | 5.59 | 2.09 | | .008 | -1.67 | 0.23 | <.001 | -11.04 | | 1.22 | <.001 | |
| CS type*Group | -0.87 | 1.61 | | .590 | | 15.53 | 1.97 | | <.001 | 14.32 | 1.61 | <.001 | -4.07 | | 2.73 | .136 | |
| Group*Phase/Trial | -0.12 | 0.35 | | .590 | | 23.82 | 2.09 | | <.001 | -1.56 | 0.23 | <.001 | -0.73 | | 1.22 | .550 | |
| CS type*Phase/Trial*Group | -0.16 | 0.70 | | .821 | | 32.97 | 4.13 | | <.001 | -2.40 | 0.46 | <.001 | -2.24 | | 2.44 | .358 | |

Experiment 1 with all participants included (Hypotheses 1a & 1b):

SCR

| Main effects and interactions | Pavlovian fear acquisition training | | | | | US-avoidance acquisition | | US-avoidance-reward | | Pavlovian extinction | | | Test | | | | |
| --- | --- | --- | --- | --- | --- | --- | --- | --- | --- | --- | --- | --- | --- | --- | --- | --- | --- |
|  | β | | SE | | *p* | β | SE | | *p* | β | SE | *p* | β | SE | | | *p* |
| CS type | 0.19 | 0.016 | | <.001 | | 0.080 | 0.014 | | <.001 | 0.084 | 0.016 | <.001 | 0.090 | | 0.026 | <.001 | |
| Phase/Trial | -0.0050 | 0.0035 | | .154 | | -0.018 | 0.015 | | .215 | -0.0011 | 0.0023 | .632 | -0.026 | | 0.011 | .025 | |
| Group | -0.058 | 0.050 | | .251 | | -0.060 | 0.047 | | .210 | -1.03 | 0.051 | .045 | -0.10 | | 0.054 | .069 | |
| CS type*Phase/Trial | 0.024 | 0.0069 | | <.001 | | 0.079 | 0.030 | | .008 | -0.0047 | 0.0045 | .305 | -0.031 | | 0.023 | .176 | |
| CS type*Group | -4.7*10^-4^ | 0.032 | | .988 | | 0.038 | 0.028 | | .175 | 0.020 | 0.031 | .522 | -0.067 | | 0.051 | .189 | |
| Group*Phase/Trial | -0.0057 | 0.0069 | | .414 | | 0.025 | 0.030 | | .397 | 0.0034 | 0.0045 | .452 | 0.025 | | 0.023 | .264 | |
| CS type*Phase/Trial*Group | 0.013 | 0.014 | | .330 | | 0.061 | 0.059 | | .305 | -1.4*10^-4^ | 0.0091 | .988 | -0.0081 | | 0.045 | .858 | |

Experiment 1 with all participants included – Additional models (Hypotheses 1c, 2a & 2b):

Expectancy ratings

| Main effects and interactions | Anticipatory fear predicted by US-avoidance during phases of US-avoidance acquisition | | | Protection from extinction | | | Protection from extinction predicted by US-avoidance in Extinction | | | | |
| --- | --- | --- | --- | --- | --- | --- | --- | --- | --- | --- | --- |
|  | β | SE | *p* | β | SE | *p* | β | SE | | | *p* |
| CS type | 41.70 | 1.17 | <.001 | 36.66 | 2.53 | <.001 | 54.16 | | 3.20 | <.001 | |
| US-avoidance | -0.29 | 0.017 | <.001 | NA | | | 0.21 | | 0.041 | <.001 | |
| CS type*US-avoidance | -0.69 | 0.026 | <.001 |  |  |  | 0.19 | | 0.077 | .012 | |
| Phase/Trial | NA | | | 23.97 | 2.53 | <.001 | NA | | | | |
| Group |  |  |  | 0.91 | 2.71 | .738 |  |  |  |  |  |
| CS type*Phase/Trial |  |  |  | 44.68 | 4.34 | <.001 |  |  |  |  |  |
| CS type*Group |  |  |  | 4.36 | 4.34 | .316 |  |  |  |  |  |
| Group*Phase/Trial |  |  |  | -5.90 | 4.34 | .175 |  |  |  |  |  |
| CS type*Phase/Trial*Group |  |  |  | -9.68 | 8.66 | .265 |  |  |  |  |  |

Experiment 1 with all participants included – Additional models (Hypotheses 1c, 2a & 2b):

SCR

| Main effects and interactions | Anticipatory fear predicted by US-avoidance during phases of US-avoidance acquisition | | | Protection from extinction | | | Protection from extinction predicted by US-avoidance in Extinction | | | |
| --- | --- | --- | --- | --- | --- | --- | --- | --- | --- | --- |
|  | β | SE | *p* | β | SE | *p* | β | SE | | *p* |
| CS type | 0.092 | 0.017 | <.001 | 0.10 | 0.044 | .019 | 0.10 | | 0.054 | .058 |
| US-avoidance | -3.21*10^-4^ | 2.47*10^-4^ | .194 | NA | | | 0.0012 | | 8.02*10^-4^ | .144 |
| CS type*US-avoidance | -0.0015 | 4.23*10^-4^ | <.001 |  |  |  | 4.19*10^-5^ | | 0.0013 | .975 |
| Phase/Trial | NA | | | 0.033 | 0.044 | .448 | NA | | | |
| Group |  |  |  | -0.11 | 0.060 | .077 |  |  |  |  |
| CS type*Phase/Trial |  |  |  | 0.057 | 0.087 | .516 |  |  |  |  |
| CS type*Group |  |  |  | 0.0044 | 0.087 | .960 |  |  |  |  |
| Group*Phase/Trial |  |  |  | -0.086 | 0.087 | .325 |  |  |  |  |
| CS type*Phase/Trial*Group |  |  |  | 0.069 | 0.17 | .691 |  |  |  |  |

Experiment 2 with all participants included (Hypotheses 1a & 1b):

US-avoidance

| Main effects and interactions | Pavlovian fear acquisition training | | | US-avoidance acquisition | | US-avoidance-reward | | Pavlovian extinction | | | Test | | |
| --- | --- | --- | --- | --- | --- | --- | --- | --- | --- | --- | --- | --- | --- |
|  | β | SE | *p* | β | SE | | *p* | β | SE | *p* | β | SE | *p* |
| CS type | NA | | | 44.40 | 3.20 | | <.001 | 36.64 | 0.94 | <.001 | NA | | |
| Phase/Trial |  |  |  | -13.08 | 1.10 | | <.001 | -1.03 | 0.14 | <.001 |  |  |  |
| Group |  |  |  | -18.53 | 4.35 | | <.001 | -25.45 | 5.02 | <.001 |  |  |  |
| CS type*Phase/Trial |  |  |  | -5.29 | 2.12 | | .013 | -1.35 | 0.27 | .046 |  |  |  |
| CS type*Group |  |  |  | 1.48 | 2.00 | | .460 | -5.16 | 1.87 | .006 |  |  |  |
| Group*Phase/Trial |  |  |  | -25.09 | 2.12 | | <.001 | 0.54 | 0.27 | .046 |  |  |  |
| CS type*Phase/Trial*Group |  |  |  | -15.78 | 4.23 | | <.001 | -0.15 | 0.54 | .787 |  |  |  |

Experiment 2 with all participants included (Hypotheses 1a & 1b):

US-expectancy

| Main effects and interactions | Pavlovian fear acquisition training | | | | | US-avoidance acquisition | | US-avoidance-reward | | Pavlovian extinction | | | Test | | | | |
| --- | --- | --- | --- | --- | --- | --- | --- | --- | --- | --- | --- | --- | --- | --- | --- | --- | --- |
|  | β | | SE | | *p* | β | SE | | *p* | β | SE | *p* | β | SE | | | *p* |
| CS type | 80.83 | 0.99 | | <.001 | | 41.50 | 1.05 | | <.001 | 30.64 | 0.90 | <.001 | 41.22 | | 1.50 | <.001 | |
| Phase/Trial | 0.59 | 0.22 | | .006 | | 0.32 | 1.11 | | .777 | -1.42 | 0.13 | <.001 | -7.36 | | 0.67 | <.001 | |
| Group | 0.94 | 0.99 | | .343 | | 7.11 | 2.99 | | .019 | 0.72 | 2.74 | .793 | -4.69 | | 2.96 | .117 | |
| CS type*Phase/Trial | 8.86 | 0.37 | | <.001 | | 5.27 | 2.16 | | .015 | -2.35 | 0.25 | <.001 | -11.74 | | 1.26 | <.001 | |
| CS type*Group | 1.74 | 1.69 | | .302 | | 22.64 | 2.03 | | <.001 | 6.26 | 1.75 | <.001 | -6.15 | | 2.82 | .030 | |
| Group*Phase/Trial | -0.0057 | 0.37 | | .988 | | 10.27 | 2.16 | | <.001 | -0.94 | 9.25 | <.001 | 0.27 | | 1.26 | .830 | |
| CS type*Phase/Trial*Group | 0.99 | 0.73 | | .179 | | 18.08 | 4.30 | | <.001 | -1.55 | 0.51 | .002 | -1.24 | | 2.53 | .625 | |

Experiment 2 with all participants included (Hypotheses 1a & 1b):

SCR

| Main effects and interactions | Pavlovian fear acquisition training | | | | | US-avoidance acquisition | | US-avoidance-reward | | Pavlovian extinction | | | Test | | | | |
| --- | --- | --- | --- | --- | --- | --- | --- | --- | --- | --- | --- | --- | --- | --- | --- | --- | --- |
|  | β | | SE | | *p* | β | SE | | *p* | β | SE | *p* | β | SE | | | *p* |
| CS type | 0.28 | 0.018 | | <.001 | | 0.089 | 0.015 | | <.001 | 0.073 | 0.015 | <.001 | 0.11 | | 0.026 | <.001 | |
| Phase/Trial | -0.011 | 0.0039 | | .005 | | -0.033 | 0.016 | | .039 | -0.0062 | 0.0022 | .004 | -0.034 | | 0.011 | .003 | |
| Group | 0.071 | 0.043 | | .100 | | 0.013 | 0.033 | | .683 | -0.051 | 0.032 | .115 | 0.025 | | 0.048 | .606 | |
| CS type*Phase/Trial | 0.033 | 0.0077 | | <.001 | | 0.0052 | 0.032 | | .871 | -0.0083 | 0.0043 | .054 | 0.0087 | | 0.023 | .703 | |
| CS type*Group | 0.085 | 0.035 | | .015 | | 0.089 | 0.030 | | .003 | 0.040 | 0.030 | .178 | 0.021 | | 0.051 | .681 | |
| Group*Phase/Trial | -0.0044 | 0.0077 | | .569 | | 0.031 | 0.032 | | .332 | -0.0072 | 0.0043 | .092 | 5.9*10^-4^ | | 0.023 | .794 | |
| CS type*Phase/Trial*Group | 0.020 | 0.015 | | .203 | | -0.013 | 0.064 | | .841 | 0.0025 | 0.0086 | .774 | 0.048 | | 0.046 | .294 | |

Experiment 2 with all participants included – Additional models (Hypotheses 1c, 2a & 2b):

Expectancy ratings

| Main effects and interactions | Anticipatory fear predicted by US-avoidance during phases of US-avoidance acquisition | | | Protection from extinction | | | Protection from extinction predicted by US-avoidance in Extinction | | | | |
| --- | --- | --- | --- | --- | --- | --- | --- | --- | --- | --- | --- |
|  | β | SE | *p* | β | SE | *p* | β | SE | | | *p* |
| CS type | 60.12 | 1.23 | <.001 | 39.96 | 2.51 | <.001 | 50.25 | | 3.30 | <.001 | |
| US-avoidance | -0.42 | 0.018 | <.001 | NA | | | 0.35 | | 0.043 | <.001 | |
| CS type*US-avoidance | -0.83 | 0.025 | <.001 |  |  |  | 0.28 | | 0.093 | .003 | |
| Phase/Trial | NA | | | 25.31 | 2.51 | <.001 |  | | | | |
| Group |  |  |  | -2.87 | 2.75 | .300 |  |  |  |  |  |
| CS type*Phase/Trial |  |  |  | 42.77 | 4.37 | <.001 |  |  |  |  |  |
| CS type*Group |  |  |  | 0.23 | 4.37 | .958 |  |  |  |  |  |
| Group*Phase/Trial |  |  |  | -3.11 | 4.37 | .477 |  |  |  |  |  |
| CS type*Phase/Trial*Group |  |  |  | -6.14 | 8.73 | .482 |  |  |  |  |  |

Experiment 2 with all participants included – Additional models (Hypotheses 1c, 2a & 2b):

SCR

| Main effects and interactions | Anticipatory fear predicted by US-avoidance during phases of US-avoidance acquisition | | | Protection from extinction | | | Protection from extinction predicted by US-avoidance in Extinction | | | |
| --- | --- | --- | --- | --- | --- | --- | --- | --- | --- | --- |
|  | β | SE | *p* | β | SE | *p* | β | SE | | *p* |
| CS type | 0.15 | 0.019 | <.001 | 0.040 | 0.038 | .288 | 0.070 | | 0.047 | .145 |
| US-avoidance | -0.0013 | 2.71*10^-4^ | <.001 | NA | | | 0.0027 | | 0.047 | <.001 |
| CS type*US-avoidance | -0.0024 | 0.00047 | <.001 |  |  |  | 0.0010 | | 0.0015 | .487 |
| Phase/Trial | NA | | | 0.19 | 0.038 | <.001 | NA | | | |
| Group |  |  |  | -0.064 | 0.052 | .224 |  |  |  |  |
| CS type*Phase/Trial |  |  |  | 0.233 | 0.074 | .002 |  |  |  |  |
| CS type*Group |  |  |  | -0.019 | 0.074 | .797 |  |  |  |  |
| Group*Phase/Trial |  |  |  | 0.11 | 0.074 | .155 |  |  |  |  |
| CS type*Phase/Trial*Group |  |  |  | 0.055 | 0.148 | .712 |  |  |  |  |
